# The Biological and Chemical Diversity of Tetramic Acid Compounds from Marine-Derived Microorganisms

**DOI:** 10.3390/md18020114

**Published:** 2020-02-15

**Authors:** Minghua Jiang, Senhua Chen, Jing Li, Lan Liu

**Affiliations:** 1 School of Marine Sciences, Sun Yat-sen University, Guangzhou 510006, China; jiangmh23@mail2.sysu.edu.cn (M.J.); lijing356@mail.sysu.edu.cn (J.L.); 2 South China Sea Bio-Resource Exploitation and Utilization Collaborative Innovation Center, Guangzhou 510006, China; 3 Southern Laboratory of Ocean Science and Engineering (Guangdong, Zhuhai), Zhuhai 519000, China

**Keywords:** tetramic acid, bioactivity, marine natural product, marine-derived microorganisms

## Abstract

Tetramic acid (pyrrolidine-2,4-dione) compounds, isolated from a variety of marine and terrestrial organisms, have attracted considerable attention for their diverse, challenging structural complexity and promising bioactivities. In the past decade, marine-derived microorganisms have become great repositories of novel tetramic acids. Here, we discuss the biological activities of 277 tetramic acids of eight classifications (simple 3-acyl tetramic acids, 3-oligoenoyltetramic acids, 3-decalinoyltetramic acid, 3-spirotetramic acids, macrocyclic tetramic acids, *N*-acylated tetramic acids, α-cyclopiazonic acid-type tetramic acids, and other tetramic acids) from marine-derived microbes, including fungi, actinobacteria, bacteria, and cyanobacteria, as reported in 195 research studies up to 2019.

## 1. Introduction

Secondary metabolites bearing a tetramic acid (pyrrolidine-2, 4-dione) motif have been isolated from various terrestrial and marine species, such as bacteria, actinobacteria, cyanobacteria, fungi, and sponges. The tetramic acid scaffold can be modified by unusual and intricate substituents to form complex, diverse chemical structures with multiple stereogenic centers. Intriguingly, an increasing number of tetramic acid products have shown a remarkable diversity of bioactivities, including antitumor, antibacterial, antifungal, and antiviral activities [1,2,3,4,5]. Due to their intricate structures and potent biological activity, natural tetramic acids have attracted a great deal of attention for their biosynthesis mechanisms, medicinal potential, and chemical synthesis in the biological, chemical, and pharmaceutical fields. Up to 2013, there were several reviews covering numerous aspects of naturally occurring tetramate products, such as isolation, biological activity, and synthesis, published by Royles [1], Ghisalberti [2], Gossauer [3], Schobert and Schlenk [4], and Ju et al. [5]. Many reviews have discussed the biosynthetic mechanisms of the PKS–NRPS biosynthesis pathways of tetramic acids in detail [2,5,6,7,8,9]. 

Marine natural products (MNPs) are considered an unexploited treasure trove of new bioactive NPs for the 21st century. Among them, marine microorganism-derived NPs have become the primary source of new MNPs, from less than 20% of newly discovered MNPs in 2006 to 57% in 2017 (based on a summary of a series of reviews “Marine Natural Products” published by Blunt and his colleagues during 2008–2019 [10,11]. While there have been no special reviews about tetramic acid compounds from marine microbes, especially in the past six years, numerous examples of new tetramate molecules from marine-derived microorganisms, and their related bioactivities, have been reported (up to 94 articles, 48% of the total 195 research articles from 1970–2019). In the current review, we focus our attention on the isolation, structural features, and biological activities of natural tetramate products isolated from marine-derived microorganisms (fungi, actinobacteria, bacteria, and cyanobacteria) reported up to September 2019. Notably, three broad groups of compounds (cytochalasins, 4-*O*-substituted derivatives (i.e., tetronates), and 2-pyridones), from putative tetramic acid-related biosynthesis pathways have been covered in numerous reviews [4,6,7,8,12,13,14,15,16] and are excluded from this review.

**Figure 1:**
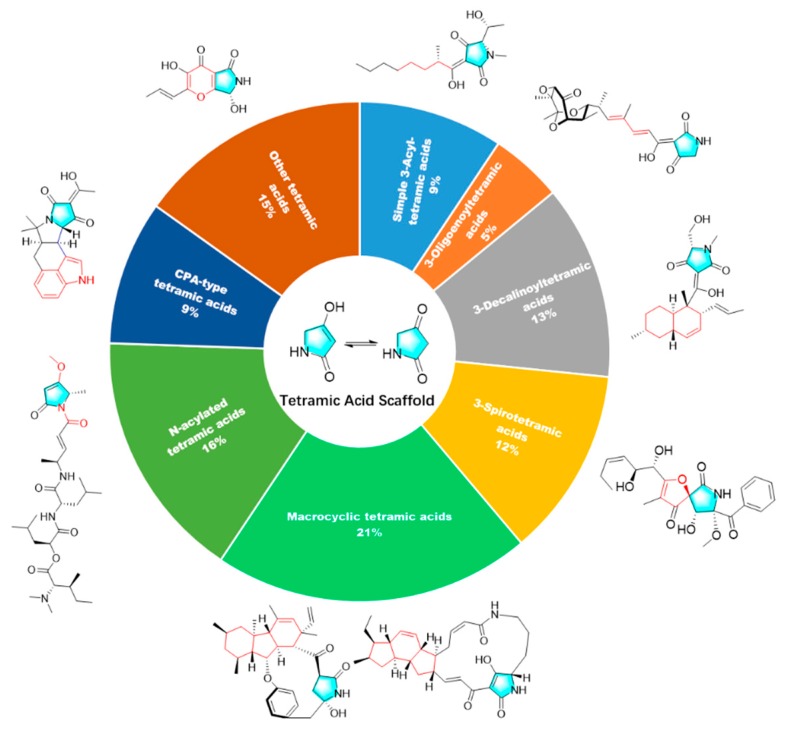
Classification of the 277 tetramic acids (TAs) from marine microorganisms into eight classes. Some examples of typical molecules belonging to these classes are illustrated: simple 3-acyl-tetramic acids (penicillenol A_1_), 3-oligoenoyltetramic acids (tirandamycin A), 3-decalinoyltetramic acids (equisetin), 3-spirotetramic acids (pseurotin A), macrocyclic tetramic acids (from left to right, ikarugamycin, GKK1032A_2_), *N*-acylated tetramic acids (symplostatin 4), α-cyclopiazonic acid (CPA)-type tetramic acids (*α*-cyclopiazonic acid), and other tetramic acids (vermelhotin). The main characteristics of each chemical class are highlighted in red.

A total of 195 research papers describing 277 tetramate compounds from marine-derived microbes were analyzed for this review (Supplementary Table S1). The assignments of a given compound to a certain category were based on their particular structural features and biogenetic pathways. The compounds were characterized into eight groups of chemical structures, as shown in Figure 1: simple 3-acyl-tetramic acids (3-ATAs), 3-oligoenoyltetramic acids (3-OTAs), 3-decalinoyltetramic acids (3-DTAs), 3-spirotetramic acids (3-STAs), macrocyclic tetramic acids (MTAs), *N*-acylated tetramic acids, α-cyclopiazonic acid (CPA)-type tetramic acids, and other tetramic acids. Furthermore, the macrocyclic tetramic acids were distributed into two subcategories: polycyclic tetramate macrolactams (PTMs) from marine actinobacteria and bacteria, and pyrrocidine tetramate alkaloids (PTAs) from marine fungi. The pie chart in Figure 1 provides deeper insight into the diversity and complexity of TAs from marine-derived microbes, revealing the complexity and diversity of molecules characterized as the dominating compounds. MTAs (21.0%) comprised the largest proportion of TAs from marine microbes, followed by *N*-acylated TAs (16.0%), 3-DTAs (13.0%), 3-STAs (12%), CPA-type TAs (9%), 3-ATAs (9%), and 3-OTAs (5%). As is known, structures can determine properties; thus, the complex and diverse structures of TAs will lead to the diversity of their bioactivities. Therefore, this review aims to give an overview of the naturally occurring tetramate products from marine-derived microbes and their biological activities, as reported in the literature until September 2019, to illustrate their biodiversity, chemical diversity, and bioactive diversity. The origins of the strains and the diversity and biological properties of the compounds, as well as the relevant publication details are also summarized (Supplementary Table S1).

## 2. Isolation, Structure, and Bioactivities of Tetramic Acid Products from Marine Microbes

### 2.1. Simple 3-acyl Tetramic Acid

**Figure 2:**
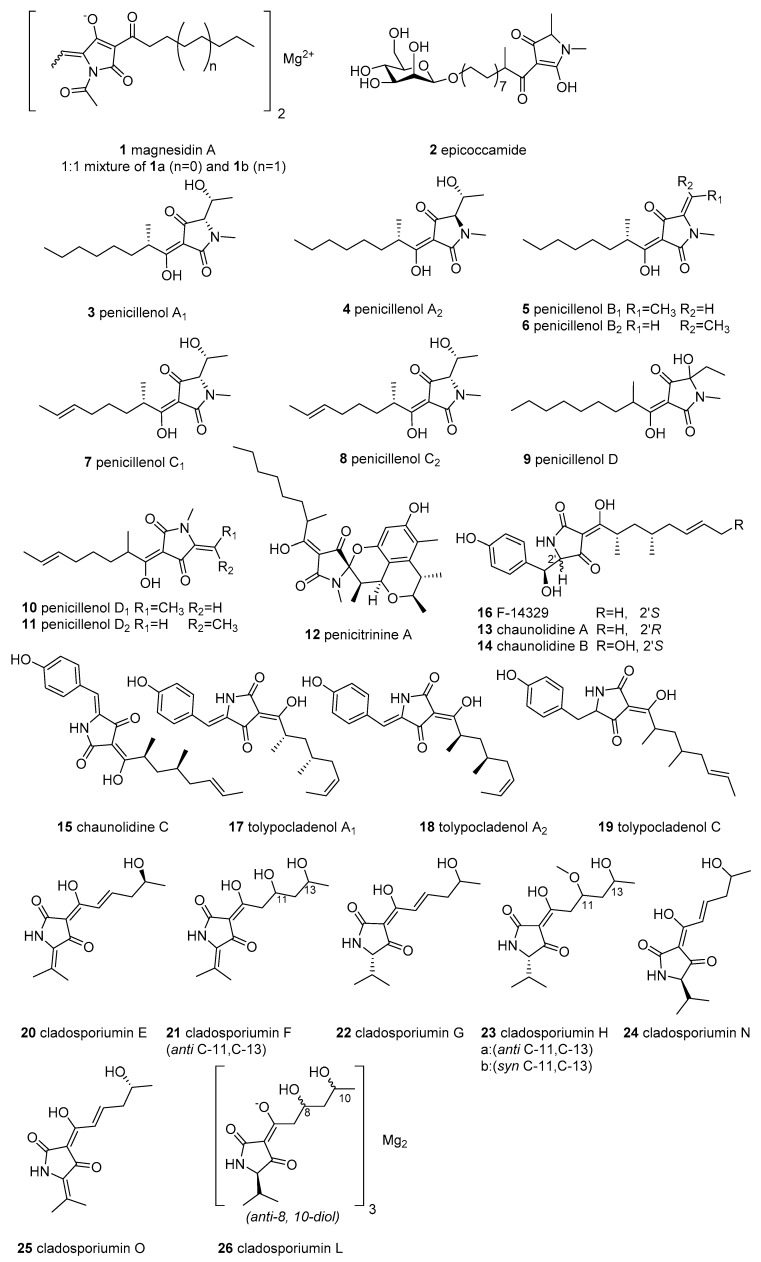
Chemical structures of simple 3-acyl tetramic acids (**1**–**26**).

Simple 3-acyl tetramic acids (3-ATAs), which contain an acyl substituent at C-3, are the most common tetramate derivatives in nature. However, only 26 simple 3-ATAs (featuring C-3-acyl-linear side chains) have been discovered from marine microorganisms (Figure 2).

Magnesidin A (**1**), a mixture of the magnesium chelates of the **1**a (3-hexanoyl) and **1**b (3-decanoyl), was first isolated in 1973 from the marine bacterium *Pseudomonas magnesiorubra* and re-isolated in 1994 from another marine bacterium, *Vibrio gazogenes*, and displayed significant activity against eight Gram-positive bacteria (MIC = 2–7 μg/mL) [17,18]. Epicoccamide (**2**), an unusual *O*-glycosylated tetramic acid with a *β*-D-mannose moiety and an aliphatic chain, was reported from a marine fungus *Epicoccum purpurascens* originating from the jellyfish *Aurelia aurita*, and was devoid of antimicrobial and cytotoxic activities [19].

The bioassay-guided investigation of the fermentation culture of *Penicillium* sp. GQ-7, which was collected from an endophytic fungus associated with the mangrove plant *Aegiceras corniculatum*, led to the isolation of six tetramic acids, penicillenols A_1_, A_2_, B_1_, B_2_, C_1_, and C_2_ (**3**–**8**) [20]. Subsequently, the penicillenol analogues penicillenol D (**9**) [21] and penicillenols D_1,_ and D_2_ (**10** and **11**) [22] were discovered from the marine sediment-derived fungi *Trichoderma citrinoviride* and *Penicillium citrinum*, respectively. The stereochemistry (**3**–**8**) of C-9 in the 3-acyl side chain was assigned as *S* by total synthesis [23]. In the cytotoxicity bioassay, compound **3** showed potent cytotoxicity against five human tumor cell lines (HTCLs) (A-549, BEL-7402, P388, HL-60, and A375) with IC_50_ values of 23.80, 13.03, 8.85, 0.76, and 12.80 μM, respectively [20,24], while **4**, **5**, and **6** displayed cytotoxicity against the HL-60 cell line, with an IC_50_ ranging from 3.20 to 16.26 μM [20]. Further, compounds **9**, **10**, and **11** showed moderate or weak cytotoxicity against A-375 (IC_50_ = 32.6 μM for **9**), A549, and HL-60 (IC_50_ = 43.5–66.5 μM for **10** and **11**) [21]. Penicillenol A_1_ (**3**) also showed cytotoxicity against cisplatin-resistant HT-29, antibacterial activity against *Staphylococcus aureu*s, and antituberculous (anti-TB) activity, with a 96.1% inhibition ratio at 10 μM [25]. In seeking anti-biofilm agents, these molecules (**3**–**8)** were re-obtained from the deep-sea fungus *Aspergillus restrictus* DFFSCS006 and were used to inhibit biofilm formation and eradicate the pre-developed biofilms of *Candida albicans* [26]. The mechanistic basis of compounds **4** and **5** is to decrease hyphal growth, thereby suppressing the transcripts of specific genes, inhibiting the expression of extracellular polymeric substance and reducing phospholipase activity [26]. The structure–activity relationships (SARs) of these penicillenols suggest that the saturation of the hydrocarbon chain at C-8 and the *trans*-configuration of the double bond between C-5 and C-6 might significantly affect their activities; further, a different configuration of C-5 is important for anti-biofilm (*R*) and antitumor (*S*) activities [20,26]. 

Penicitrinine A (**12**), bearing a unique 5-spiro tetramate skeleton and considered to be a Diels–Alder reaction product of compound **3** and citrinin, was purified from a strain of *P. citrinum* (also producing compounds **3**–**6** [24]), and displayed antiproliferative activities on multiple HTCLs, especially human malignant melanoma cell A-375 [27]. The mechanism of action was via inducing apoptosis by regulating Bcl-2 and Bax secretion and inhibiting cell metastasis through suppressing MMP-9 activity and upregulating its specific inhibitor TIMP-1 [27].

Four simple 3-ATAs, chaunolidines A–C (**13**–**15**) and a known F-14329 (**16**) were isolated from the marine fungus *Chaunopycnis* sp. (CMB-MF028) associated with a pulmonate false limpet *Siphonaria* sp. [28]. All have the capability to form metal chelates nonselectively, though only **16** exhibits cytotoxic activity and was previously reported to inhibit the absorption of neutral lipids in mice [28,29]. The three tetramate analogues tolypocladenol A_1_, A_2_, and C (**17**–**19**) were obtained from sponge-derived *Tolypocladium geodes* sp. MF458 using the “one strain many compounds” (OSMAC) method and do not exhibit cytotoxicity [30]. Seven 3-ATAs (cladosporiumins E–H, N–O, and L as an Mg complex) (**20**–**23**, **24**–**25,** and **26**) were isolated from *Cladosporium* sp. SCSIO z0025 [31] and *Cladosporium sphaerospermum* EIODSF 008 [32] was derived from deep-sea sediment. 

### 2.2. 3-Oligoenoyltetramic Acids

**Figure 3:**
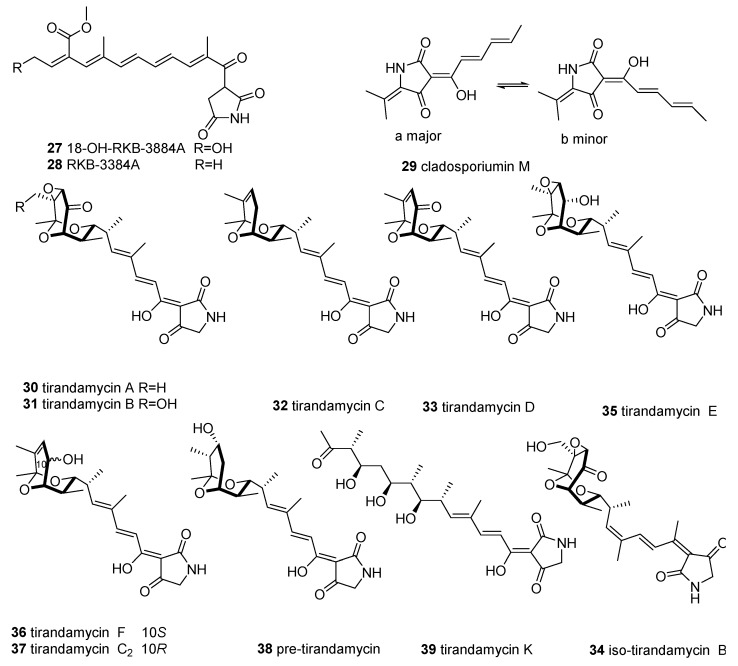
Chemical structures of 3-oligoenoyltetramic acids (**27**–**39**).

To date, only 13 members of 3-oligoenoyltetramic acids (3-OTAs) (Figure 3) possessing a 1-oxopentadienyl substituent at C-3 in the tetramate ring have been discovered from marine microorganisms—three of them from fungi and ten of them from actinobacteria *Streptomyces*.

The fermentation broth of the mangrove soil-derived fungus *Aspergillus* sp. OUCMDZ-1914 yielded two 3-oligoenoyltetramic acids, RKB-3884A (**27**) and its analogue 18-OH-RKB-3884A (**28**). Molecule **27** showed potent inhibition of the H1N1 influenza virus (IC_50_ = 116.2 μM), equal to the positive control ribavirin (IC_50_ = 138.1 μM) [33]. Another 3-OTA, cladosporiumin M (**29**), was isolated from the deep-sea-derived fungus *Cladosporium sphaerospermum* EIODSF 008, and was devoid of cytotoxic and antibacterial activities [32].

Tirandamycins (TAMs) are a subgroup of *Streptomyces*-derived 3-dienoyltetramic acid antibiotics that exhibit broad biological activities, such as bacterial RNA polymerase inhibition [34], inhibition of mitochondria oxidative phosphorylation [35], and effects on the futalosine pathway [36]. By screening new MNPs with anti-vancomycin-resistant *Enterococcus faecalis* (VRE) activity, researchers have reported tirandamycins A–D (**30**–**33**), isolated from the marine environmental isolate *Streptomyces* sp. 307-9, among which **30** showed the highest activity (MIC 2.25 µM) [37]. The SARs of these tirandamycins suggest that the C-10 ketone and C-11/C-12 epoxide confer increased potency, but this effect can be attenuated by the hydroxy group at C-18 [37]. Compounds **30** and **31** are considered as the main antibacterial constituents of marine *Streptomyces* sp. [38,39]. Moreover, tirandamycin B (**31**) was reported to be a new lead scaffold for anti-filarial activity, as it can selectively inhibit *Brugia malayi* AsnRS (BmAsnRS) (IC_50_ = 30 μM) and efficiently kill adult *B. malayi* parasite (IC_50_ = 1 μM) in vitro without general cytotoxicity to human hepatic cells [40,41]. An assessment of the anti-*VRE* activity and BmAsnRS inhibition of TAMs revealed that these bioactivities were strongly dependent on the structure of the dioxabicyclo[3.3.1]nonane ring unit [37]. Isotirandamycin B (**34**), together with tirandamycins A and B, were identified from the marine-derived *Streptomyces* sp. SCSIO 41399 and displayed potent bacteriostatic activity against *Streptococcus agalactiae* (MIC = 5.7–11.5 µM) [42]. Using a biosynthetic approach, tirandamycins C (**32**), E (**35**), F (**36**), C2 (**37**), and pre-tirandamycin (**38**), were isolated from the genetically engineered strains of Streptomyces sp. 307-9 and Streptomyces sp. SCSIO1666 [43,44,45]. Similarly, the first linear 7,13;9,13-diseco-tirandamycin derivative tirandamycin K (**39**) was obtained from a mutant strain (△tamI) of marine *Streptomyces* sp. 307-9 [46]. Furthermore, studies on the biological activity of **39** and other TAMs confirmed that the bicyclic ketal ring of TAMs is the key pharmacophore [46].

### 2.3. 3-Decalinoyltetramic Acids

**Figure 4:**
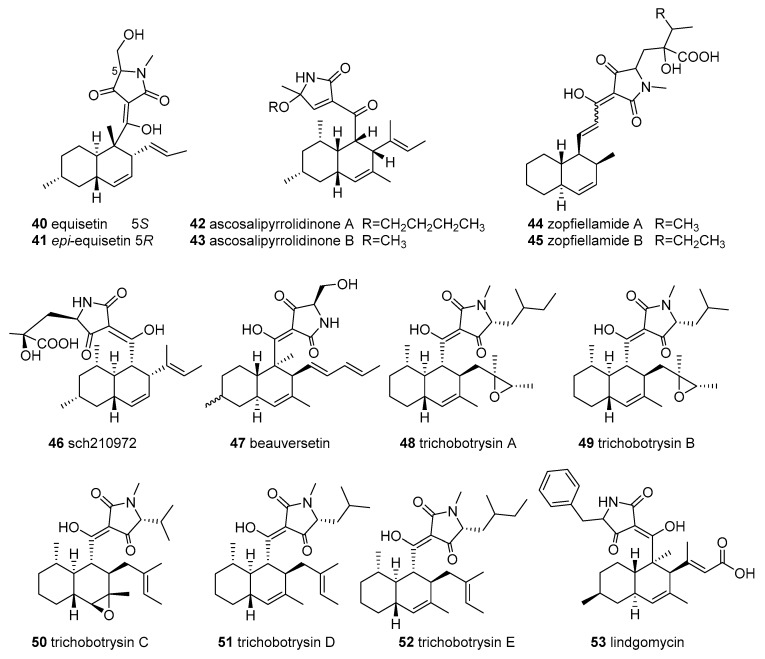

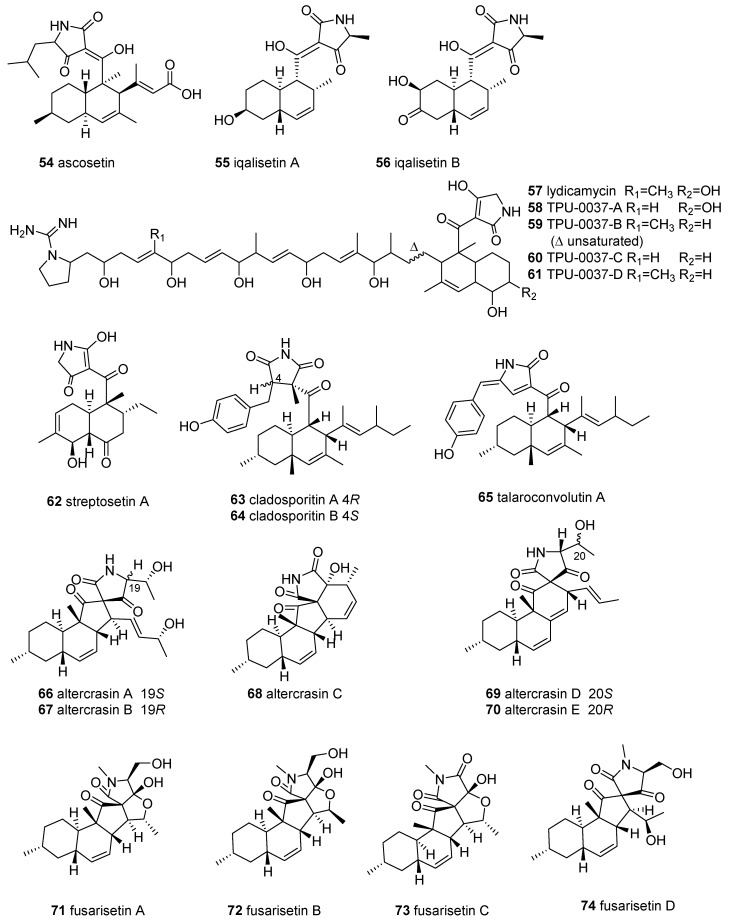
Chemical structures of 3-decalinoyltetramic acids (**40**–**74**).

The class of 3-decalinoyltetramic acids (3-DTAs) derived from microorganisms features a tetramate unit at position *N*-1 connected to H or CH_3_, and C-3 connected to “decalin” with multiple chiral centers. Up to 35 members of 3-DTAs (Figure 4) have been uncovered from marine fungi and actinobacteria.

The typical 3-DTA, equisetin (**40**) and epi-equisetin (**41**), were isolated initially from terrestrial Fusarium genera and displayed various biological activities, such as antimicrobial, anti-HIV, cytotoxicity, and phytotoxicity activities [1]. These two molecules were also later isolated from the marine-derived fungi *Fusarium* sp. 152 and *F. equiseti* D39 [47] and displayed potent anti-phytopathogenic bacterial and fungal activities [47]. Notably, equisetin (**40**) exhibited potent anti-methicillin-resistant *Staphylococcus aureus (MRSA*) activity (MIC = 1 µg/mL, equivalent to vancomycin) and antimicrobial activities against *Pseudomonas syringae* and *Rhizoctonia cerealis* (MIC = 1.1 and 8.4 µM, respectively) superior to the positive control and could be exploited as a potential antimicrobial drug candidate [48].

Ascosalipyrrolidinones A and B (**42** and **43**), possessing a rare *cis*-decalin scaffold, were isolated from an obligate marine fungus, *Ascochyta salicorniae*, collected from the green alga *Ulva* sp. [49]. Compound **42** displayed moderate antiplasmodial activity towards the *Plasmodium falciparum* strains K-1 and NF-54, antimicrobial activity, the inhibition of tyrosine kinase p56^lck^, and significant antiprotozoal activity against *Trypanosoma cruzi* and *T. brucei*, as well as cytotoxic activity against rat skeletal muscle myoblast cells and mouse peritoneal macrophages [49]. Another two 3-decalinoyltetramic acids, zopfiellamides A and B (**44** and **45**), were isolated from the culture broth of a marine soil-derived ascomycete Zopfiella latipes CBS 611.97 [50]. These metabolites **(44** and **45)** displayed antifungal activity against the yeasts Nematospora coryli and *Saccharomyces cerevisiae* at MIC = 2 μg/mL and were devoid of cytotoxicity against four HTCLs [50]. One of the compounds, **44,** displayed moderate antibacterial activity against ten bacterial strains (MIC = 2–10 μg/mL), which was about five times more active than that of **45**, showing that the extra methyl group of zopfiellamides influences antibacterial properties [50]. The seaweed-derived fungus *Microdiplodia* sp. yielded sch210972 (**46**), which could inhibit human leucocyte elastase (HLE), with an IC_50_ value of 1.04 µg/mL, and revealed moderate inhibition of the growth of *Bacillus megaterium* [51]; **46** can also be considered a chemokine receptor CCR-5 inhibitor, with an IC_50_ of 79 nM [52]. Another marine-sponge-derived fungus, Beauveria bassiana, afforded a 3-DTA named beauversetin (**47**), which exhibited moderate cytotoxicity against a panel of six cell lines, with a mean IC_50_ = 3.09 μg/mL for a monolayer assay, but it was devoid of antimicrobial activity [51]. A class of new 3-DTAs, trichobotrysins A–E (**48**–**52**), were discovered from the culture of a deep-sea-derived fungus *Trichobotrys effuse* DFFSCS021 [53]. Among them, compounds **48**, **49,** and **51** showed significant selective inhibition of the proliferation of carcinoma KG-1*α* HTCL (IC_50_ = 5.44, 8.97, and 6.16 µM) and prominent antiviral activity towards HSV-1 (IC_50_ = 3.08, 9.37, and 3.12 µM) [53]. Lindgomycin (**53**), with its unique 5-benzyl-3-decalin-tetramate skeleton, and ascosetin (**54**) were obtained from two marine-derived *Lindgomycetaceae* strains and showed moderate antibiotic activities against six Gram-positive bacteria, including *MRSA* and two pathogenic fungi with IC_50_ = 2.2–17.8 μM [54,55]. Chemical investigation of the crude extracts from the marine sediment-derived fungus *Tolypocladium* sp. yielded two new 3-DTAs, iqalisetins A and B (**55** and **56**), which lacked the tested activities [56]. 

The fermentation broth of a marine actinomycete *Streptomyces platensis* (TP-A0598) provided lydicamycin (**57**) and four new analogues, TPU-0037 A–D (**58**–**61**) possessing octahydrodecalin skeletons [57]. The lydicamycins (**57**–**61**) showed significant bioactivity against five Gram-positive bacteria (including MRSA), with MIC values in the range of 0.39–12.5 μg/mL [57,58]. Among them, molecule **60** displayed the most potent bioactivity, while **59** showed the lowest level of bioactivity, indicating that the C14–C15 olefin may diminish antibacterial activity [57]. During screening, human class III histone deacetylase (SIRT) inhibitors from the marine actinomycete streptosetin A (**62**) were discovered from the broth of Streptomyces sp. CP13-10 and displayed weak inhibitory activity towards yeast Sir2p, human SIRT1, and SIRT2, with IC_50_ values of 2.5, 3.7, and 4.5 μM, respectively [59].

Recently, a chemical analysis of the culture broth of the mangrove-derived fungus *Cladosporium* sp. HNWSW-1 resulted in the discovery of two new succinimide-containing derivatives, cladosporitins A and B (**63** and **64**), together with talaroconvolutin A (**65**) [60]. Compound **64** showed moderate cytotoxicity against three HTCLs, with IC_50_ values from 25.6 to 41.7 µM, whereas **65** exhibited moderate cytotoxicity towards two HTCLs (IC_50_ = 14.9 and 26.7 µM), as well as significant inhibitory activity against α-glycosidase (IC_50_ = 78.2 µM) [60]. 

Altercrasins and fusarisetins are 3-DTA derivatives with a unique structure, some of which exhibited unusual bioactivities. Altercrasin A (**66**), a novel decalin derivative with spirotetramic acid, was reported from a strain of *Alternaria* sp. OUPS-117D-1, originally associated with the sea urchin *Anthocidaris crassispina*, and displayed moderate inhibitory activity against three HTCLs [61,62]. Other studied metabolites of this fungal strain, altercrasins B–E (**67**–**70**), were obtained in 2019 [62]. Two pairs of stereoisomers **66**/**67** and **69**/**70** were characterized by an unusual 6/6/5/5 tetracyclic ring system, while **68** was identified as a 6/6/5/6/5 pentacyclic ring [62]. These isolated compounds **66**–**70** had moderate or potent cytotoxicity against three leukemia HTCLs (IC_50_ = 6.1–61 μM), two of which, **69** and **70**, bear a diene moiety (C-6 to C-8) and exhibited significant cytotoxicity against HL-60, with IC_50_ values of 6.1 and 6.2 μM, respectively, similar to that of 5-fluorouracil (IC_50_ = 4.5 μM) [62]. Using the bioassay-LCMS-^1^H NMR-screening technology, four 3-DTA derivatives, namely fusarisetins A–D (**71**–**74**), were discovered from a marine-derived fungus *Fusarium equiseti* D39 and displayed phytotoxicity [47]. Interesting, fusarisetins A–C possess a rare carbon skeleton with a 6/6/5/5/5 pentacyclic ring system, while fusarisetin D is the first-discovered fusarisetin with an unusual 6/6/5/5 tetracyclic ring framework. It was reported that fusarisetin A also has acinar morphogenesis inhibitory activity [63]. Interestingly, fusarisetins A and B are considered as a novel class of potent cancer migration inhibitors with a new mechanism of action [64,65]. The SAR of the fusarisetins revealed that the decalin motif, serine amino acid, and C-18 oxygen are critical to the biological profile of fusarisetins [64]. 

### 2.4. 3-Spirotetramic Acids

**Figure 5:**
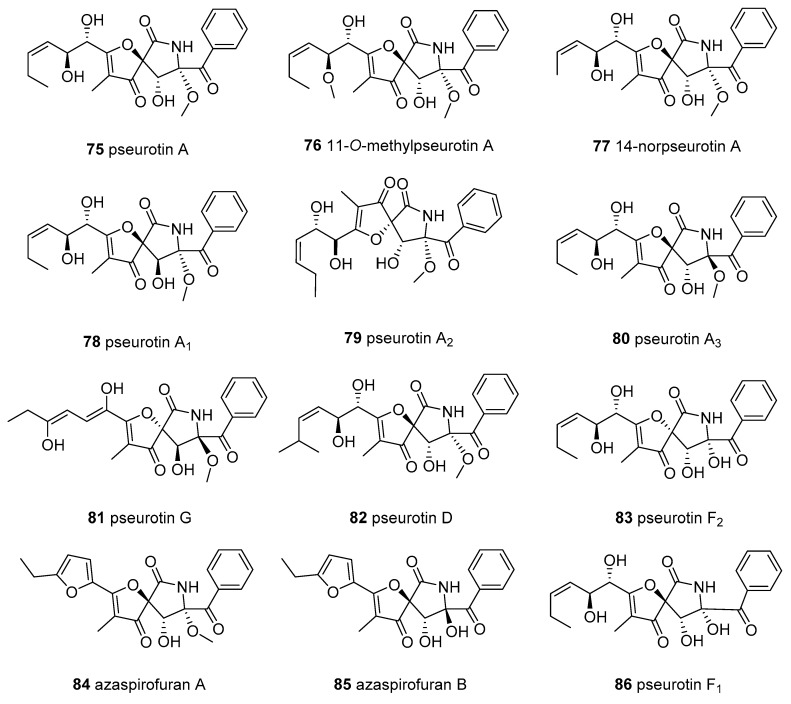

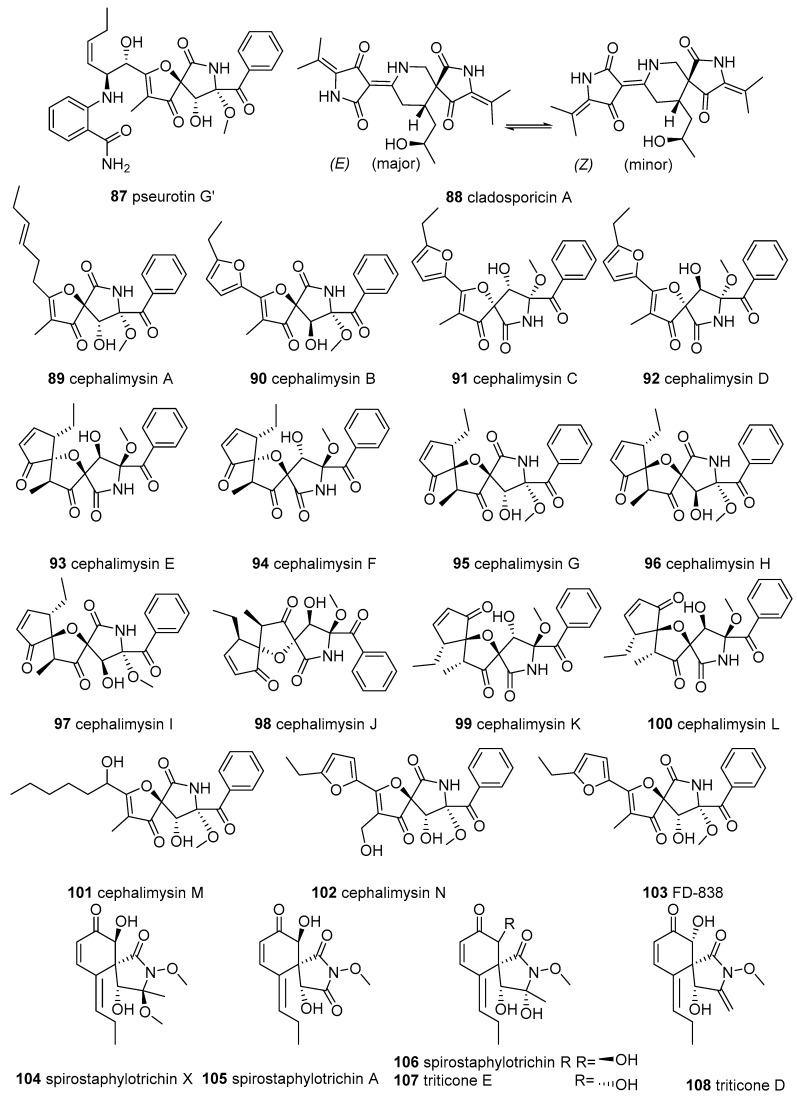
Chemical structures of 3-spirotetramic acids (**75**–**108**).

Thirty-four members of 3-spirotetramic acids (3-STAs) (Figure 5) were isolated from marine fungi. The 3-STAs principally consisted of 3-spirofuranone-lactam TAs (FD-838, 11 pseurotins, 14 cephalimysins, and 2 azaspirofurans) and 3-STAs with hexatomic rings (three spirostaphylotrichins, two triticones, and cladosporicin A). The 3-spirofuranone-lactam TAs, possessing a 1-oxa-7-azaspiro[4.4]nonane core with a phenyl ketone and C-6-aliphatic appendages, rarely occur in natural sources.

Pseurotin A (**75**) was initially isolated from the broth of a fungal strain Pseudeurotium ovalis Stolk in 1976 [66]. In recent years, pseurotin A and ten pseurotin-related analogues have been discovered from marine fungi *Aspergillus fumigatus* [67], *Aspergillus sydowii* [68]*, Aspergillus* sp. [69], and *Phoma* sp. [70]. It is worth mentioning that the marine bacteria *Bacillus* sp. FS8D can also yield **75** [71]. Interestingly, pseurotin A showed extensive bioactivities, including monoamine oxidase inhibitory activity [72], apomorphine-antagonistic activity [73], chitin synthase inhibitory activity [74], the induction of cell differentiation [75], nematicidal activity [76], immunosuppressive activity [77], antiparasitic and cytotoxicity [78], antibacterial activity [79,80], antioxidant activity [81], and osteoporosis inhibition through suppressing reactive oxygen species levels [82]. 

Using a yeast halo assay as a bioassay-guided fractionation of marine-derived *A. fumigatus* resulted in the identification of 11-*O*-methyl pseurotin A (**76**), which selectively inhibited the Hof1 deletion strain [67]. The fermentation broth of the marine driftwood-derived fungus *A. sydowii* PFW1-13 yielded pseurotin A (**75**) and 14-norpseurotin A (**77**) [68]. Compound **77** displayed significant antimicrobial activities against *Escherichia coli, Bacillus subtilis*, and *Micrococcus lysodeikticus*, with MICs of 3.74, 14.97, and 7.49 µM [68], moderate antiparasitic activity against *Leishmania donovan* and Plasmodium *falciparum*, and cytotoxicity against MCF-7 and U937; **77** also significantly induced the neurite outgrowth of rat pheochromocytoma cells (PC12) at 10.0 µM [78,83]. Using the bioassay-guided method to investigate another fungus, *A. fumigatus* WFZ-25, which is associated with marine holothurian, this group obtained two new pseurotins, pseurotin A1 and A2 (**78** and **79**), as well as **75 [84]**, which were also re-isolated from the marine fish-derived *A. fumigatus* OUPS-T106B-5 and resulted in a structural revision of pseurotin A2, as shown in **79 [85]**. When screening for compounds with cytotoxicity and anti-inflammatory activities, two new 3-STAs, pseurotins A3 and G (**80** and **81**), as well as their analogues pseurotins D **(82),** F2 **(83),** A**(75)**, A1 **(78)**, and A2 **(79)**, were identified from the marine fungus *Phoma* sp. NTOU4195, separated from the edible red alga *Pterocladiella capillacea* [70]. According to the bioassays, compound **81** revealed moderate antiangiogenic activity by inhibiting tube formation in human endothelial progenitor cells, with an IC_50_ value of 16.7 μM; compounds **78–81** displayed moderate anti-inflammatory inhibitory activity against NO production using LPS-induced RAW 264.7cCells (IC_50_ = 34.5-62.5 μM, aminoguanidine 24.7 μM) [70]. In addition, pseurotins D (**82**) displayed an apomorphine-antagonistic effect [73], as well as antiparasitic and cytotoxicity affects [78], and pseurotins F2 (**83**) demonstrated apomorphine-antagonistic activity [86] and chitin synthase inhibition [74].

Screening for antitumor agents, the marine-sediment-derived fungus *Aspergillus sydowii* D2-6 was found to produce two new 3-STAs, named azaspirofurans A and B (**84** and **85**), which feature a new furan ring instead of the long linear side chain of pseurotin [87]. In vitro cytotoxicity experiments have demonstrated that **85** has moderate cytotoxicity toward A549 HTCL (IC_50_ = 10 μM) [87]. Six years later, azaspirofuran B (**85)** and pseurotin F1 (**86**) were re-obtained from a marine jellyfish-derived fungus *A. fumigates* [88]. In recent years, seven known 3-STAs, **75**, **76**, **79**, **83**–**86,** were isolated from the marine *Aspergillus fumigatus* MR2012, associated with Red Sea sediment, using zebrafish embryos and larvae in an attempt to discover promising compounds from marine microorganisms that may have in vivo antiseizure activity [89]. Based on a series of experiments (including the larval zebrafish pentylenetetrazole seizure experiment, electrophysiological analysis, and ADMET assessment) among them, **79** and **84** were demonstrated to be lead antiseizure compounds and possible new antiseizure therapeutics [89]. A new pseurotin derivative, pseurotin G’ (**87**), together with 11-*O*-methyl pseurotin A (**76**), was discovered from the co-culture of the fungus *A. fumigatus* MR2012 and the bacterium *Streptomyces leeuwenhoekii* C34 [90]. 

The *E*/*Z* mixture, cladosporicin A (**88**), containing a rare 2,7-diazaspiro[4.5]decane-1,4-dione skeleton conjugated with a tetramate moiety, was identified from *Cladosporium sphaerospermum* SW67 in association with the marine fungus *Hydractinia*, and displayed weak cytotoxicity against four HTCLs [91].

Fifteen cephalimysins and their analogue FD-838, containing a spiroheterocyclic *γ*-lactam motif and six chiral centers, were found in the marine *Aspergillus fumigatus*. The fermentation broth of *A. fumigatus* OUPS-T106B-5 provided a class of 3-STAs consisting of cephalimysins A–L (**89**–**100**) and FD-838 (**103**) [92,93,94,95]. Among them, compounds **90**–**92** are diastereomers of 103 [93], which was first reported in a patent as being able to induce differentiation of leukemic cells, as well as to have antibacterial and antifungal activities [81,96]. Cytotoxicity experiments revealed that cephalimysins **89**–**100** (except for **90**) and **103,** have moderate cytotoxic activity against several HTCLs [92,93,95], particularly **89,** which is cytotoxic against the murine P388 and human HL-60 cell lines (IC_50_ = 15.0 and 9.5 μM) [92], with **96** and **97** displaying cytotoxicity against the L1210 leukemia cell line (IC_50_ = 12.8 and 14.3 μM) [95] and **96** and **95** exhibiting potent cytotoxicity towards the KB epidermoid carcinoma cell line (IC_50_ = 7, 11.1 μM, equal to that of 5-fluorouracil IC_50_ = 8.5 μM) [95]. Recently, cephalimysins M and N (**101** and **102**), together with **89** and **103**, were identified as co-metabolites of the marine fungus *A. fumigatus* CUGBMF17018, while neither of them displayed antimicrobial activities [97].

A subgroup of the fungal 3-STA derivatives with a 6-membered carbocyclic motif, spirostaphylotrichin X (**104**), and three related analogues, spirostaphylotrichins A and R as well as triticone E (**105**, **106**, and **107**), were identified as metabolites of the marine alga-derived fungus *Cochliobolus lunatus* SCSIO41401 [98]. Compounds **105–107** showed weak or inactive anti-influenza virus (IAV) activity, while **104** displayed a noticeable inhibitory effect against multiple IAVs (IC_50_ = 1.2–5.5 μM) by inhibiting polymerase PB2 protein activity and interfering with the production of its progeny’s viral RNA, thus representing a new type of potential lead compound for anti-IAV therapeutics [98]. Another analogue, triticone D (**108**), was isolated from the marine sediment-derived *Westerdykella dispersa*, and found to lack antibacterial and cytotoxic properties [99]. 

### 2.5. Macrocyclic Tetramic Acids 

**Figure 6:**
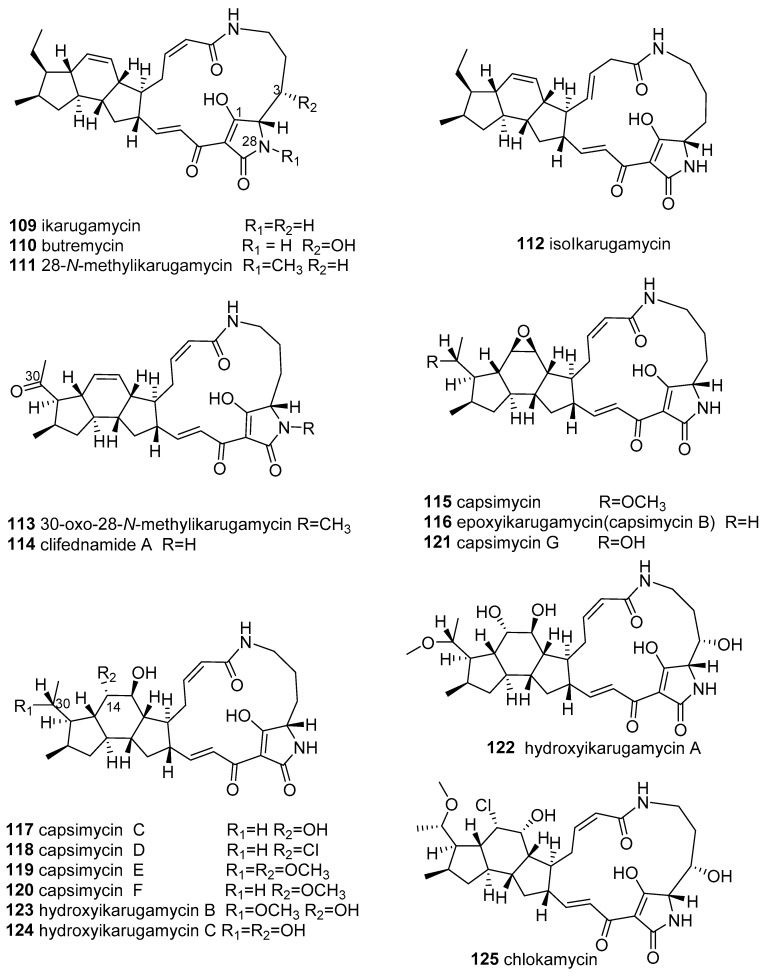

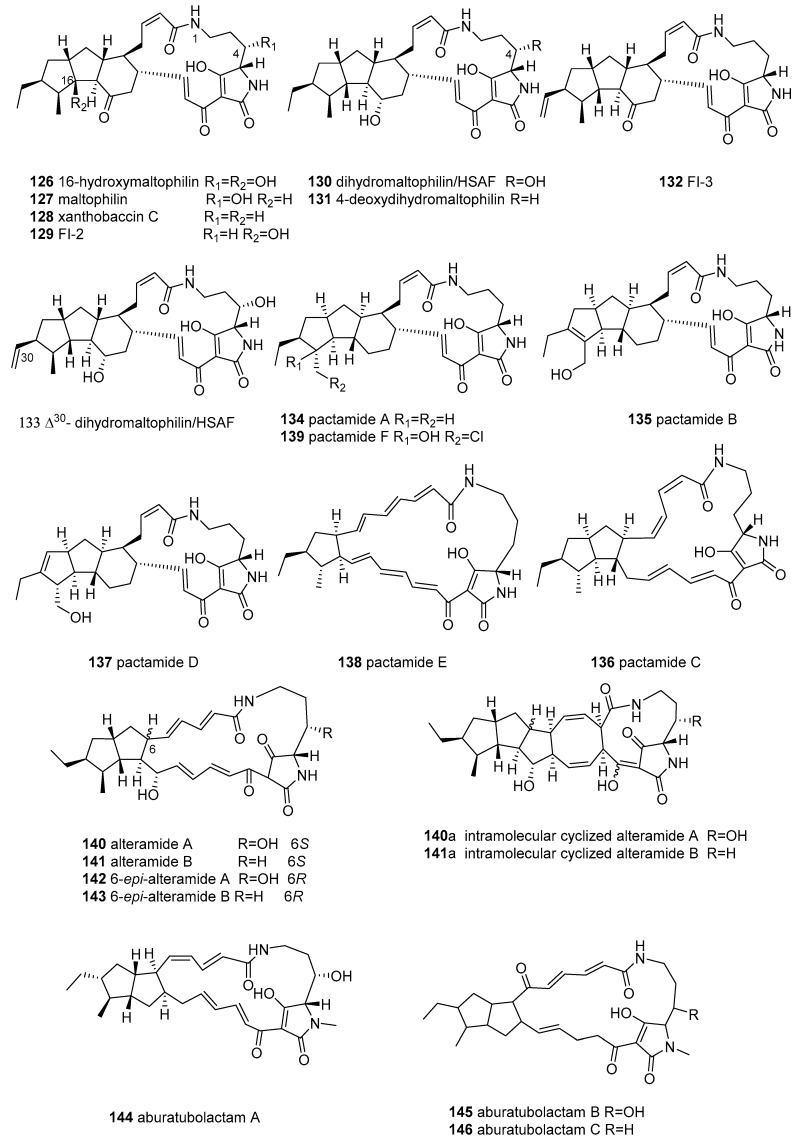
Chemical structures of macrocyclic tetramic acids-polycyclic tetramate macrolactams (**109**–**146**).

**Figure 7:**
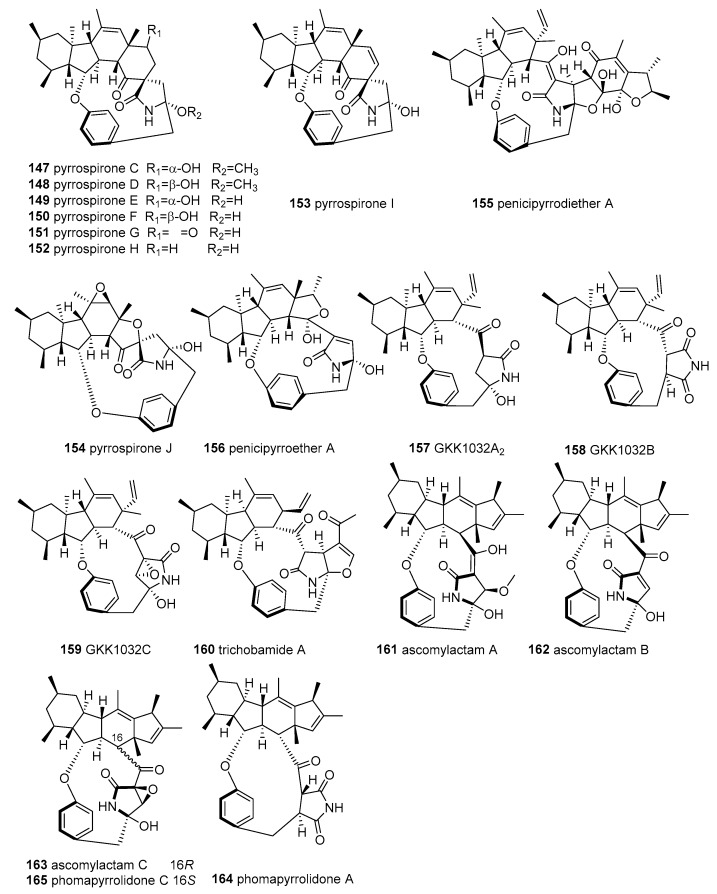
Chemical structures of macrocyclic tetramic acids-pyrrocidine tetramate alkaloids (**147**–**165).**

Macrocyclic tetramic acids (MTAs) have become compounds of great importance and interest, due to their complex structures and unique bioactivities in the field of natural products. More than 57 members of MTAs collectively constitute the major source of TAs from marine microorganisms. MTAs can be categorized into two subgroups: polycyclic tetramate macrolactams (PTMs) (Figure 6) from marine actinobacteria and bacteria, and pyrrocidine tetramate alkaloids (PTAs) (Figure 7) from marine fungi. Structurally, PTMs are composed of a polycyclic carbocycle (5, 5/5, 5/5/6 or 5/6/5 ring system) and a 16-membered macrolactam core fused with a TA moiety at C-3 [9,100]. PTAs are very rare in nature, featuring a polycyclic carbocycle (6/5/6, 6/5/6/6, or 6/5/6/5 ring system), a 12- or 13-membered macrocyclic-integrated 1,4-disubstituted phenyl and tetramate or its analogue moieties at C-3. MTAs originated from a conserved PKS/NRPS pathway, while tetramate polyene is considered to be the initial precursor of PTMs and tyrosine–nonaketide is the precursor of PTAs [8]. Moreover, the PKS module was used for the macrocyclic TA backbone assembly [101], and the NRPS domain incorporated L-ornithine [102] and L-tyrosine [8].

#### 2.5.1. Polycyclic Tetramate Macrolactams (PTMs)

There are 17 members of PTMs featuring a 5/6/5 tricyclic ring system (collectively called 5/6/5-PTMs or ikarugamycins) found in marine actinomyces, including ikarugamycins, butremycin, capsimycins, clifednamides, and chlokamycin (**109**–**125** in Figure 6).

Ikarugamycin (**109**), the first-described 5/6/5 PTM possessing a unique asymmetric-hydrindacene skeleton, was reported in 1972 [103] from *Streptomyces phaeochromogenes* var. *ikaruganensis.* Subsequently, its absolute configuration was elucidated by Hirata in 1977 [104]. Compound **109** was reported to exhibit various biological activities, possessing potent antiprotozoal [103], antibacterial (Gram-positive bacteria, including *MRSA*) [103,104,105,106], antifungal [106], and antitumor activity [107,108,109,110], inhibiting the uptake of oxidized low-density lipoproteins in macrophages [111] and inhibiting both the downregulation of HIV-1 Nef-induced CD4 on the cell surface [112] and clathrin-dependent endocytosis [113].

In subsequent years, many ikarugamycin-related structures have been continually isolated from marine-derived actinomycetes. Butremycin (**110**), a 3-hydroxylated ikarugamycin, was reported in 2014 from the new Ghanaian mangrove river-sediment-derived actinomycete *Micromonospora* sp. K310, representing the first example of a microbial producer of ikarugamycins other than the *Streptomyces* species; however, it only displayed fragile antibacterial activity (MIC ≥ 50 μg/mL) [114]. The following year, three new ikarugamycins, 28-*N*-methylikarugamycin (**111**), iso-ikarugamycin (**112**), and 30-oxo-28-*N*-methylikarugamycin (**113**), as well as **109,** were obtained from marine-sediment-derived *Streptomyces zhaozhouensis* CA-185989 [106]. Ikarugamycins **111**, **112,** and **109** showed significant anti-*MRSA* (MIC 1–4 μg/mL) activity and antifungal properties against *Candida albicans* and *Aspergillus fumigatus* (MIC = 2–8 μg/mL), while **113** displayed weak or no antimicrobial activity (MIC ≥ 32–64 μg/mL) [106]. Ikarugamycins **109**, **111**, **113**, and clifednamide A (**114**), were re-discovered from a marine sponge-associated novel actinomycete *Streptomyces zhaozhouensis* (strain MCCB267) using a cytotoxicity-guided strategy [108]. All compounds (**109**, **111**, **113**, and **114**) displayed promising cytotoxic activity against NCI-H460 lung carcinoma cells (IC_50_ = 1.43–16.26 μg/mL) by binding with DNA and disrupting the cell cycle to induce apoptotic stimuli leading to cell death in the G1 or S phase [108].

Capsimycin (**115**), bearing an *O*-methoxy group at the C-30 of ikarugamycin epoxide (**116**), was first reported as an antifungal agent from *Streptomyces* sp. C49–87 in 1979 [115]. In 2003, ikarugamycin epoxide (later termed capsimycin B) (**116**), along with **109,** were discovered from *Streptomyces* sp. Tü 6239 [105] and showed moderate antibacterial activity and cytotoxicity [105,110]. Utilizing biosynthetic technology, capsimycins C-G (**117**–**121**) and three known PTMs (**109**, **115**, and **116**) were identified from the marine mangrove-derived *Streptomyces xiamenensis* 318 [109]. Among them, the known PTMs (**109**, **115**, and **116**) exhibited strong antiproliferation activities against pancreatic carcinoma (IC_50_ = 1.30–3.37 μM), with negligible cytotoxicity towards normal cells at the same concentrations [109]. In addition, compound **115** inhibited six pancreatic carcinoma cells (IC_50_ = 2.2–7.59 μM), with weak cytotoxicity against normal cells (IC_50_ = 9.64 μM) [109]. Capsimycins **117**–**120** were significantly less actively cytotoxic than **109**, **115**, and **116**, emphasizing the importance of the C-13/C-14 double bond and epoxide ring for cytotoxicity [109]. Under a genomics-guided approach, cultivation of the marine sediment-derived *Streptomyces* sp. SCSIO 40060 led to the isolation and characterization of three new PTM analogues, hydroxyikarugamycins A–C (**122**–**124**) and four known PTMs, **109**, and **115**–**117** [110]. Unfortunately, compounds **122**–**124** were devoid of bioactivities [110]. 

A new chlorinated 565-PTM chlokamycin (**125**), together with **109,** was isolated from the culture broth of the marine-derived *Streptomyces* sp. MA2-12 [116]. Compound **125** moderately inhibited the growth of Jurkat cells and HCT116 cells, with IC_50_ values of 24.7 and 33.5 μM, respectively [116].

Twelve members of PTMs bearing the 5/5/6 ring system (collectively known as 5/5/6-PTMs), including maltophilins, xanthobaccins, HSAFs, FIs, and pactamides A, B, D, and F, were isolated from marine actinomyces (**126**–**135, 137,** and **139** in Figure 6).

The fermentation broth of the marine-derived *Actinoalloteichus cyanogriseus* WH1-2216-6 yielded a new 5/5/6-PTM named 16-hydroxymaltophilin (**126**) and five known analogues, maltophilin (**127**), xanthobaccin C (**128**), frontalamide precursor FI-2 (**129**), dihydromaltophilin/HSAF (heat-stable antifungal factor) (**130**), and 4-deoxydihydromaltophilin (**131**) [117]. In cytotoxic assays, the 5/5/6-PTMs **126**–**128** and **130**–**131** showed significant cytotoxicity against six HTCLs, with IC_50_ values of 0.1–9.7 μM. Among them, compound **126** revealed the most selective cytotoxicity against seven HTCLs, with IC_50_ = 4.5-9.7 μM (selectivity index = 24.3–51.4) [117]. Compounds **127**, **128,** **130**, and **131** also displayed antifungal activity (*Aspergillus fumigatus* AF293), with MIC = 1.56–25.0 μg/mL [117]. However, compounds **130** and **127** were most effective in their antifungal activities, with MIC values of 1.56 and 3.125 μg/mL. It was indicated that the 3-OH and 14-OH group of 5/,5/6-PTMs possibly contributed to antifungal activity, while the 16-OH group decreased bioactivity [117]. In the same year, chemical and genetic profile analyses of the marine cone-snail-associated *Streptomyces* CMB-CS038 yielded four 5/5/6 PTMs, **130,** and three minor co-metabolites, **128**, as well as the frontalamide precursor FI-3 (**132**) and a new HSAF derivate, △^30^-dihydromaltophilin (**133**) [100]. Notably, compound **130** is a potent broad-spectrum antifungal agent with a novel mechanism of action and observable cytotoxicity [100,118,119].

The activation of the silent PTM gene clusters of the *Streptomyces pactum* SCSIO 02999 by genome-mining led to the production of six new PTMs, 5/5/6-PTMs (pactamide A, B, D, and F) (**134**, **135**, **137**, and **139**), 5/5-PTM (pactamide C **136**), and 5-PTM (pactamide E **138**), which (except for compounds **135** and **137**) displayed potent or moderate cytotoxic activity against the four HTCLs of IC_50_ = 0.24–8.7 μM [120]. Among these compounds, **134** exhibited the most active cytotoxic activity, with IC_50_ values of 0.24–0.51 μM, whereas **135** and **137** showed weak cytotoxicity, with IC_50_ = 14.50–26.15 μM, suggesting that the presence of a double bond in the A ring of the 5/5/6 ring system significantly decreased their cytotoxicity [120].

Alteramides are a family of PTMs containing a 5/5 ring system fused to the macrolactam. Alteramides A and B (**140** and **141**) were obtained from the marine-sponge-associated bacterium *Alteromonas* sp. by the Kobayashi group in 1992 [121]. Their corresponding isomers, 6-*epi*-alteramides A and B (**142** and **143**), were sourced from the coral-associated *Pseudoalteromonas* sp. OT59 [122] by microbial MALDI-imaging mass spectrometry coupled with a molecular network strategy and were used to revise the original stereochemistry of alteramides, which were originally isolated from the *Streptomyces albus* J1074 in 2014 [123]. Alteramides **142** and **143** were responsible for the observed antifungal activity of this strain when grown in the dark, although they were inactivated by light through photoinduced intramolecular [4+4] cycloaddition to generate the hexacyclic products **140**a and **141**a [121,122]. Further, **140** exhibited in vitro cytotoxicity against P388, L1210, and KB cells, with IC_50_ values of 0.1, 1.7, and 5.0 µg/mL, respectively [121], while **141** showed no cytotoxicity, indicating that the presence of the C-25-hydroxyl group led to the abolishment of antiproliferative activity [121]. 

Aburatubolactams A–C (**144**–**146**) were isolated as the metabolites of a mollusk-associated *Streptomyces* sp. SCRC-A20 by the Uemura group in 1996 and 1998 [124,125]. Aburatubolactams (**144**–**146**) inhibited the TPA-induced superoxide anion generation by human neutrophils (IC_50_ 26, 6.3, 2.7 µg/mL, respectively), which is related to inflammation, cancer, and aging [125]. Additionally, **144** showed cytotoxicity, antimicrobial activity, and the inhibition of superoxide generation [125,126]. Compound **146** was cytotoxic to five leukemia and lymphoma HTCLs, inducing apoptosis, with IC_50_ values of 0.3–1.9 µg/mL [124].

#### 2.5.2. Pyrrocidine Tetramate Alkaloids (PTAs)

Pyrrocidine tetramate alkaloids (PTAs) (**147**–**165** in Figure 7) form a class of complicated and changeable MTAs, and were recently isolated from marine fungi. These PTAs, bearing a polycyclic unit (6/5/6, 6/5/6/6, or 6/5/6/5 ring system), a 12- or 13-membered macrocyclic integrated 1,4-disubstituted phenyl, and a TA (or its analogue framework at C-3), are very unusual in the natural product field.

A cytotoxicity-guided chemical investigation of the marine-derived fungus Penicillium sp. ZZ380 resulted in the isolation of seven rare new pyrrospirones C–I (**147–153**) [127] featuring a 6/5/6/6 fused ring system, minor penicipyrrodiether A (**155**) [128], and GKK1032B (**158**). Compound **151** showed potent cytotoxicity against four glioma cells, with IC_50_ values of 1.06–8.52 µM, while being devoid of antibacterial activity [128]. PTAs **147**–**150**, **152**–**153**, and **155** showed moderate cytotoxicity, with IC_50_ values of 7.44–29.10 μM, and antibacterial activity against both *MRSA* and E. coli, with MICs of 2.0–34.0 μg/mL (**147**, **150,** and **153**: MICs = 2.0–5.0 μg/mL) [127,128]. It is worth noting that **155** was the first example of a hybrid of phenol A fused to the GKK1032 skeleton via the addition of a five-membered ether ring and displayed anti-*MRSA* activity (MIC = 5.0 μg/mL) [128]. Through further chemical investigation by the OSMAC method, this strain yielded two novel PTAs with a unique 6/5/6/5 polycyclic fusion, penicipyrroether A and pyrrospirone J (**154** and **156**) [129]. Compound **154** displayed potent selective antiproliferative activity against human glioma U87MG and U251 cells (IC_50_ = 1.64–5.50 µM), equivalent to doxorubicin, and potent antibacterial activity against *MRSA* and E. coli, with MIC = 1.7–3.0 µg/mL [129].

A new member of the GKK1032 family, GKK1032C (**159**), and four known analogues, GKK1032A2 (**157**), GKK1032B (**158**), and pyrrospirones E and F (**149** and **150**), were identified from the culture of the mangrove endophytic fungus, *Penicillium* sp. CPCC 400817. Among them, compounds **159** and **157** exhibited potent antibacterial activity against *MRSA*, with MIC values of 1.6 and 3.2 µg/mL [130]. The GKK1032 family possesses unique structural features, with 12- or 13-membered macrocyclic ether-containing 1,4-disubstituted phenyl and TA components, such as *γ*-lactam or succinimide scaffolds, and a rare tricarbocyclic system in polyketides [130].

From the marine ascidian-derived fungus *Trichobotrys effuse* 4729, our research group recently obtained a novel pyrrocidine alkaloid, trichobamide A (**160**), with an unprecedented tetrahydro-5*H*-furo[2,3-b]pyrrol-5-one moiety. This molecule (**160**) significantly inhibited the proliferation of U251 and SNB19 glioma cell lines by inducing apoptosis in human glioma cells through the P53/Bax/Bcl-2 pathway [131]. The fermentation of the mangrove endophytic fungus *Didymella* sp. CYSK-4 afforded three new natural products, ascomylactams A–C (**161**–**163**) and the known analogues phomapyrrolidones A and C (**164** and **165**) [132], whose configurations were revised as **164** and **165**, bearing an unusual 6/5/6/5 tetracyclic skeleton fused with a 12- or 13-membered macrocyclic motif [133]. In the cytotoxic assay, **161** and **163** showed moderate cytotoxicity against six HTCLs (MDA-MB-435, MDA-MB-231, SNB19, HCT116, NCI-H460, and PC-3), with IC_50_ values in the range of 4.2−7.8 μM [133], while **162** and **165** displayed weak cytotoxicity against six HTCLs, with IC_50_ = 4.5−29 μM [133], and **165** exhibited weak antitubercular activity, with MIC 5.2-13.4 μg/mL in vitro [132].

### 2.6. N-acylated Tetramic Acids

**Figure 8:**
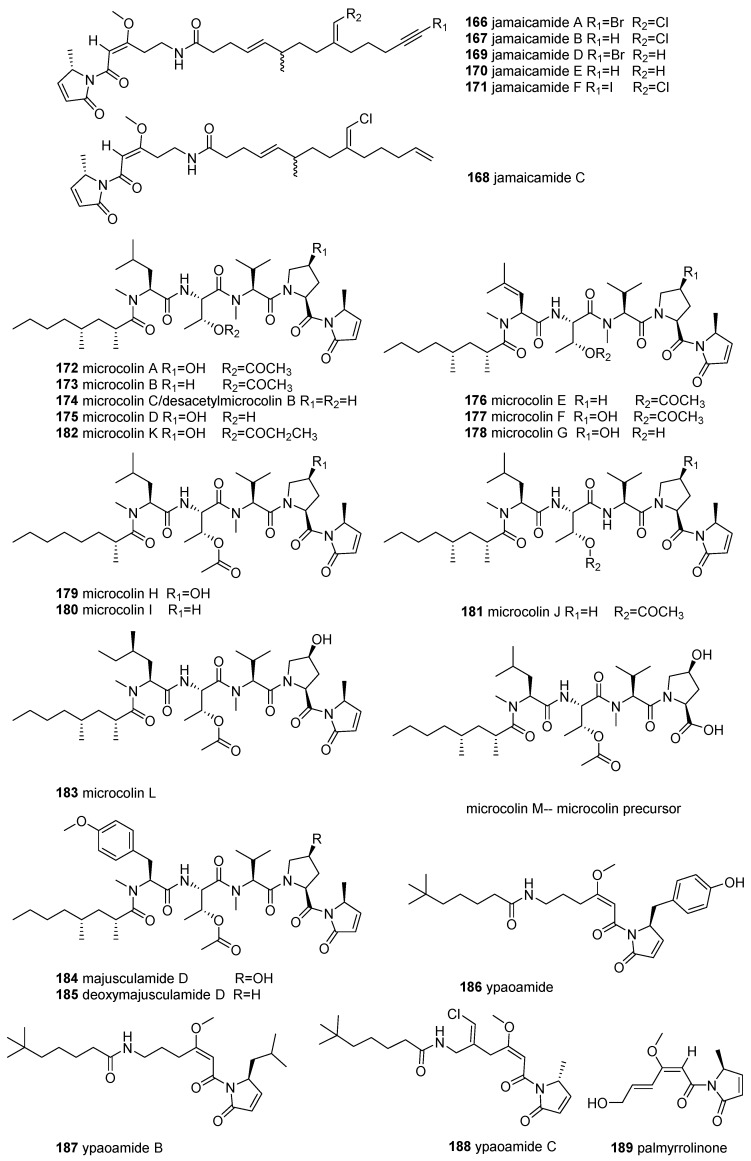

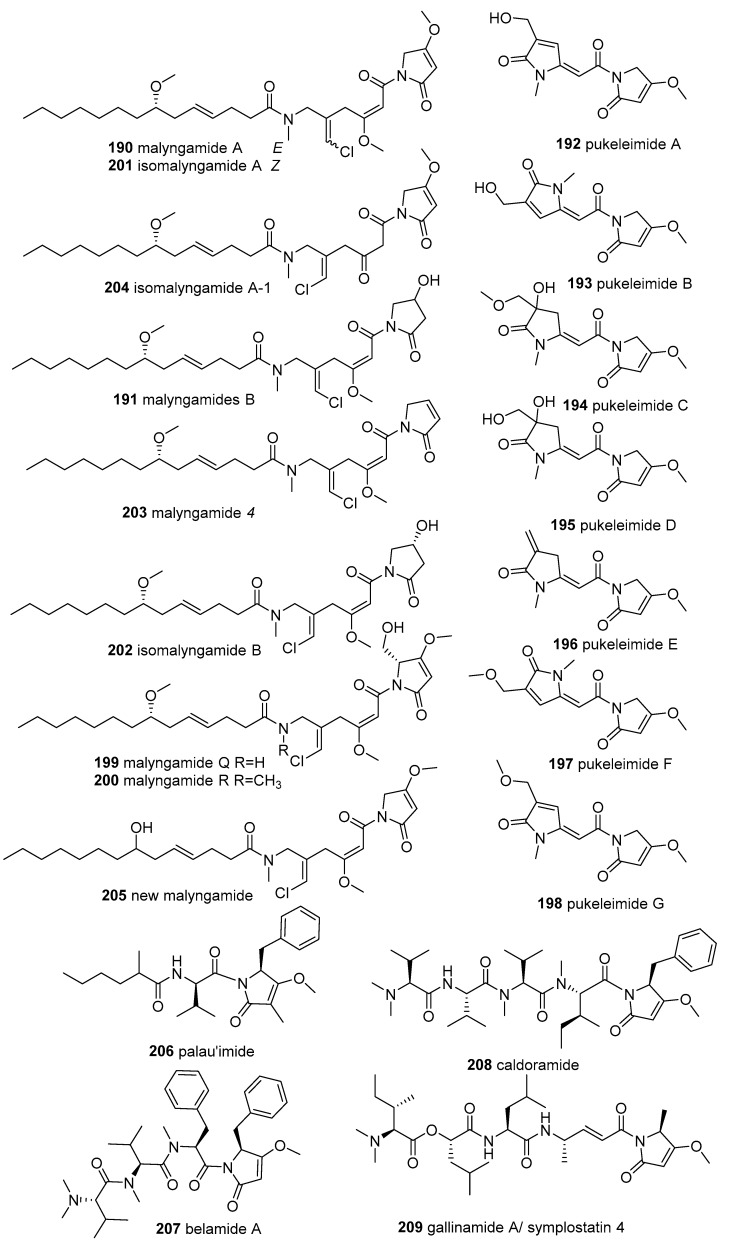
Chemical structures of *N*-acylated tetramic acids (**166**–**209**).

The class of *N*-acylated tetramic acids (44 compounds, **166**–**210** in Figure 8) contains the tetramate components, *N*-acyl-3-pyrrolin-2-one (6 jamaicamides, 11 microcolins, 2 majusculamides, 3 ypaoamides, and malyngamide 4) or *N*-acyl-4-methoxy-3-pyrrolin-2-ones (6 malyngamides, 7 pukeleimides, belamide A, caldoramide, symplostatin 4, and palau’imide), or their related derivatives, commonly found as linear lipopeptides in the marine cyanobacterium *Moorea producens (*formerly *Lyngbya majuscula*). Although *N*-acylated TAs like dolastatin 15 were also discovered from marine animals (e.g., sea hares feeding on cyanobacterium), the actual producer has always been considered to be their symbiotic cyanobacterium [134].

Jamaicamides, a class of linear hybrid NRPS/PKS neurotoxic lipopeptides with two peptide bonds and halogen, were isolated from marine *M. producens* collected in Hector Bay, Jamaica by the Gerwick group [135,136]. Using a bioassay-guided strategy, jamaicamides A–C (**166**–**168**), were isolated and found to display sodium-channel-blocking activity at 5 μM and cytotoxicity against the H-460 and Neuro-2a cell lines (LC_50_ = 15 μM), two of which (**167** and **168**) showed neurotoxic activity (100% lethality at ≤10 ppm after 90 min) in a goldfish toxicity assay [135]. Three other new analogues, jamaicamides D–F (**169**–**171**), were discovered from this strain using an orthogonal natural product workflow, containing LC-MS/MS molecular networking and OSMAC approaches [136]. Some of the jamaicamides (**166**, **167,** and **171**) showed concentration-dependent antagonism of an increase in neuronal [Ca^2+^]i/[Na^+^]i induced by veratridine (IC_50_ = 1.82–6.88 μM, and 1.1–3.6 μM, respectively) [136]. In comparison, compound **166,** as a sodium/calcium channel blocker in neocortical neurons, was approximately two to three times more active than compounds **167** and **171** [136].

Microcolins A and B (**172** and **173**), immunosuppressive lipopeptides, were first reported as metabolites of *L. majuscula* in Venezuela [137]. These two metabolites (**172** and **173**) and desacetylmicrocolin B (also termed microcolin C) (**174**) were also isolated from an active fractionation of *L. polychroa* in Florida [138]. Compounds **172** and **173** displayed potent immunosuppressive activity in a two-way murine mixed lymphocyte reaction (EC_50_ = 1.5 and 42.7 nM, TC_50_ = 22.6 and 191.0 nM) [137]. Recently, nine new linear lipopeptides, microcolins E–L (**176**–**183**) and their precursor microcolin M, together with the four known microcolins A–D (**172**–**175**), were isolated from marine *M. producens* using bioassay-guided and LC–MS/MS molecular networking approaches [139]. Structurally, microcolins E–G bearing unusual 4-methyl-2-(methylamino) pent-3-enoic (Mpe) acid units and microcolin L featuring 2-amino-4-methylhexanoic acid (*N*-Me-homoisoleucine) motifs are peptides that rarely occur in nature [139]. Additionally, microcolin M is the precursor of microcolins without an *N*-acyl-3-pyrrolin-2-one moiety [139]. Microcolin B (**173**) and D (**175**) were shown to be potential inhibitors of LFA-1/ICAM-1-mediated cell adhesion, with IC_50_ values of 0.15 and 0.9 µM, respectively [140]. At the same time, microcolins **172**–**174** were found to have significant inhibition of the growth of HT-29 and IMR-32 HTCLs (IC_50_ = 0.28–14 nM) [138]. The molecule **172** displayed antiproliferative and immunosuppressive effects on lymphocytes, with IC_50_ values in the nanomolar range in vitro, whose inhibitory activity was time-dependent and reversible without showing a reduction in cell viability [141]. These metabolites (**176**–**182**), along with three semisynthetic derivatives, 3,4-dihydromicrocolins, A, B and D, showed significant cytotoxicity against H-460 lung HTCL, with IC_50_ values ranging from 6 nM to 5.0 μM ( **172**, **175**, **177**, **179**, and **181,** with IC_50_ values of 6, 75, 37, 47, and 69 nM, respectively) [139]. SAR revealed that a hydroxyl group at the C-4 of proline and a double bond in the Mdp (5-methyl-1,5-dihydro-2H-pyrrol-2-one) moiety are critical for cytotoxicity [139].

Investigation of the active fraction of marine *L. majuscula* yielded two cytotoxic lipopentapeptides, majusculamide D (**184**) and deoxymajusculamide D (**185**), in 1988 [142]. Recently, **184** was re-discovered from a marine *Moorea* sp., and its absolute configuration was determined by total synthesis [143]. Compound **184** exhibited selective and potent in vitro cytotoxicity toward pancreatic (PANC-1), glioblastoma (U251N) (IC_50_ = 0.32 and 36.8 nM, respectively) and HepG2 HTCLs (IC_50_ = 1.40 μM) [143]. 

Ypaoamide (**186**), a lipopeptide with a feeding deterrent, was isolated from a marine cyanobacterial assemblage composed of *Schizothrix calcicola* and *L. majuscula* in 1996 [144]. Recently, biochemical studies on marine *Okeania* sp. collected in Okinawa produced two new analogues, ypaoamides B and C (**187** and **188**), which stimulated glucose uptake in a dose-dependent and insulin-independent manner in cultured L6 myotubes [145]. Furthermore, the effect of **188** on glucose uptake was found to occur by activation of the AMP-activated protein kinase (AMPK) pathway regulating cellular metabolism, suggested to be a potential therapeutic candidate for the treatment of Type 2 diabetes mellitus (T2DM) [145].

Palmyrrolinone (**189**), the only non-peptide *N*-acylated TA, was reported from a marine cyanobacterial assemblage consisting of cf. *Oscillatoria* and *Hormoscilla* spp. obtained from Palmyra Atoll and displayed potent molluscicidal activity against *Biomphalaria glabrata* (LC_50_ = 6.0 μM) [146].

Two chlorine-containing lipopeptides, malyngamides A and B (**190** and **191**), were described as the constituents of shallow water varieties of marine *M. producens* collected at Kahala Beach, Hawaii by Moore et al. in 1978 [147,148]. The same workers subsequently isolated seven closely related nontoxic compounds, pukeleimides A–G (**192**–**198**), lacking the fatty acid side chain and chlorine atoms of the **190** analogues from the same strain [149,150]. Malyngamide Q and R (**201** and **202**) [151] and isomalyngamides A and B (**199** and **200**) [152], a new subtype of malyngamide with different geometrical stereochemistry at C-6 (*Z*-chloromethylene)**, were purified from marine *L. majuscula* from Madagascan and Hawaiian waters, respectively. A bioassay-directed fractionation of the active fractions of a strain of *M. producens* derived from the Red Sea resulted in the isolation of a new malyngamide 4 (**203**), along with malyngamides A and B. Compounds **203** and **191** revealed moderate cytotoxicity against three HTCLs (A549, HT29, and MDA-MB-231,) (IC_50_ = 40–60 µM) [153]. (*Z*)-malyngamides **199** and **200** showed lethal toxicity to crayfish at less than 0.5 mg/kg [152]. Subsequently, a new (*Z*)-malyngamide, named isomalyngamide A-1 (**204**), along with **199**, was obtained from a Taiwanese *L. majuscula* [154]. Compounds **204** and **199** displayed potential in suppressing breast cancer cell (MDA-MB-231) migration, with nanomolar IC_50_ values of 337 and 60 nM, and blocked cell proliferation, with micromolar IC_50_ values of 12.7, and 2.8 μM, by inactivating the expression of focal adhesion kinase (FAK), *p*-FAK, Akt, and *p*-Akt through the *β*-1 integrin-mediated antimetastatic pathway [154]. It was indicated that the C-12’ enol methyl ether group of **204** was essential for its cytotoxicity against breast HTCLs [154]. A new malyngamide (**205**)—the first report of a malyngamide with a hydroxy group at C-7 of the fatty acid portion—as well as **199** and **200**, were discovered in Hawaiian *M. producens* [155]. The bioactivity of **205**, showing very weak cytotoxicity against L1210 and lethal toxicity to shrimp, was approximately 10–100 times weaker than that of **199** and **200**, suggesting that the methoxy group at C-7 of the fatty acid section was the important pharmacophore of the malyngamide [155].

Palau’imide (**206**), with an *N*-acyl-4-methoxy-3-methyl-pyrrolin-2-one unit, was isolated from a marine *Lyngbya* sp. NIH309 collected in the Palau region and was cytotoxic to KB and LoVo cells (IC_50_ = 1.4, and 0.36 μM) [156]. Belamide A (**207**), a highly methylated linear tetrapeptide analogue of dolastatins 10 and 15, was discovered in the Panamanian marine cyanobacterium Symploca sp. [157]. Compound **207** was found to be cytotoxic to MCF-7 and HCT-116 HTCLs, with IC_50_ values of 1.6 and 0.74 μM, respectively [157]. Further, **207** also displayed classic tubulin-destabilizing antimitotic characteristics by depolymerizing the microtubule network in the A-10 cell lines at 20 μM [157]. A new linear pentapeptide, caldoramide (**208)**, sharing a structural similarity to **207** and dolastatins 10 and 15, was isolated from the marine cyanobacterium *Caldora penicillata* (syn. *Phormidium penicillatum*) collected at Florida [158]. Compound **208** was cytotoxic to HCT116 colorectal cancer cells (IC_50_ = 3.9–8.6 μM) modified in oncogenic KRAS and hypoxia-inducible factor (HIF) pathways, which are related to angiogenesis, cell growth, apoptosis, and metastasis, suggesting that this compound can act as an indirect HIF inhibitor [158].

In 2009, the cyanobacterial linear lipodepsipeptide symplostatin 4 (Sym4) (**209**) [159] and gallinamide A (**209**) [160], containing a methylmethoxypyrrolinone (MMP) moiety, were independently isolated from *Symploca* sp. and *Schizothrix* sp., respectively. Subsequently, the total syntheses of both compounds revealed that they are indeed identical [160,161]. Subsequent biological evaluations of **209** and three synthetically generated N-terminal diastereoisomers demonstrated their potent antimalarial properties: potent antimalarial activities against the *Plasmodium falciparum* 3D7 strain (IC_50_ = 37–104 nM), similar to that of the positive control, chloroquine (IC_50_ = 17.8 nM) [160]. Compound **209** was also moderately activated in inhibiting the chloroquine-resistant strain of *P. falciparum* W2 (IC_50_ = 8.4 μM) [162]. Compound **209** also displayed moderate cytotoxicity against mammalian Vero cells (IC_50_ = 10.4 μM), HeLa cervical carcinoma cells (IC_50_ = 12 μM), and HT-29 colon adenocarcinoma cells (IC_50_ = 53 μM); surprisingly, the lack of cytotoxicity toward NCIH460 lung tumors or neuro-2*α* mouse neuroblastoma cell lines at 16.9 μM indicated that **209** could be considered as a promising lead antimalarial hit [159,162]. Furthermore, compound **209** was demonstrated to induce the G2 cell cycle arrest at a high micromolar concentration, which is related to microtubule-disrupting effects [162]. Notably, compound **209** did not cause the lysis of red blood cells (RBCs), even at high concentrations (>25 mM) [161], indicating that its antiparasitic effect was not due to the permeabilization of the RBC membrane. Compound **209** also potently and selectively inhibited the human cysteine protease cathepsin L (IC_50_ = 5.0 nM) through a covalent and irreversible mechanism [163]. The sym4-treatment of *P. falciparum*-infected RBCs led to the generation of a swollen food vacuole phenotype and a reduction in parasitemia at an EC_50_ of 0.7 μM [164]. Further studies of **209** and its derivatives revealed that **209** acts as a nanomolar inhibitor of the P. falciparum falcipains (FPs) in infected RBCs by inhibiting the hemoglobin degradation pathway and indicating its unusual MMP unit as the critical pharmacophores [164]. 

### 2.7. α-Cyclopiazonic acid (CPA)-type Tetramic Acids

**Figure 9:**
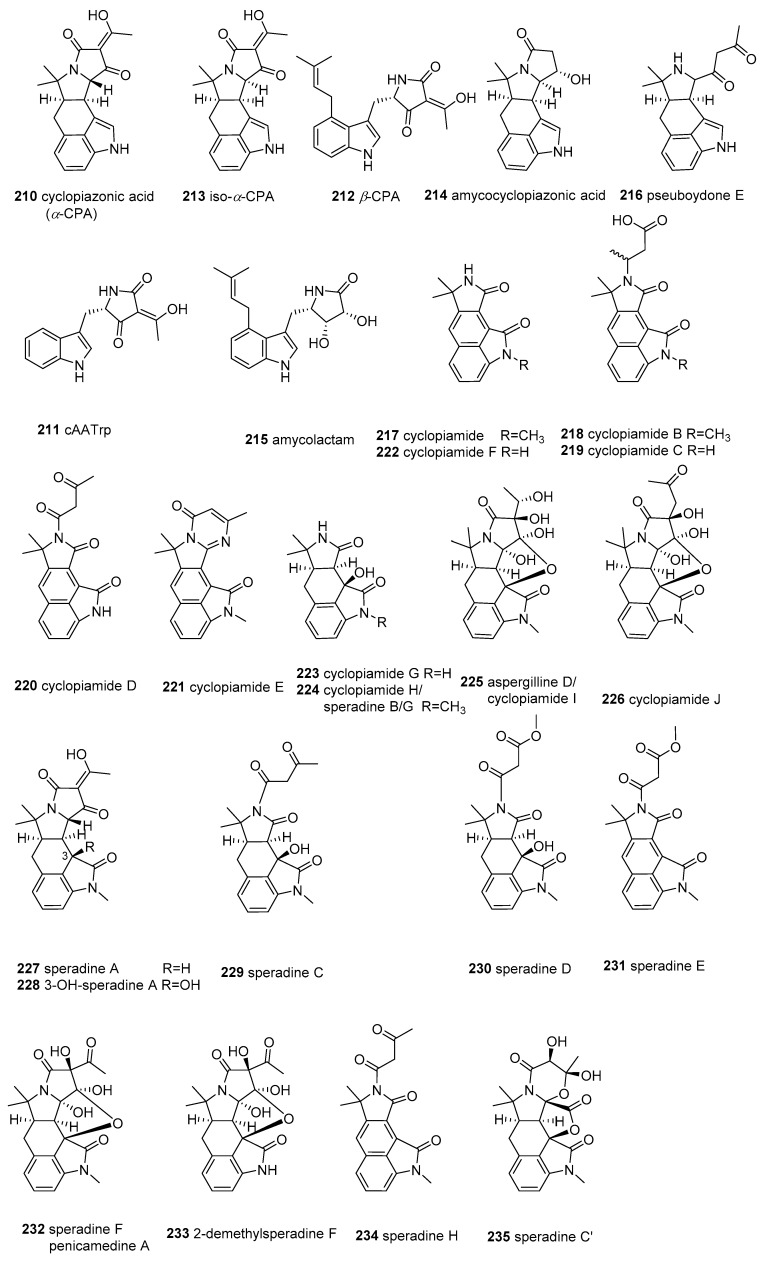
Chemical structures of CPA-type tetramic acids (**210**–**235).**

Cyclopiazonic acid (*α*-cyclopiazonic acid, *α*-CPA) (**210**) is a potentially severe mycotoxin that possesses an indole-hydrindane-tetramate unit and is produced by many fungal species in the Ascomycta genera *Penicillium* and *Aspergillus* [165]. Compound **210** was biosynthesized from three precursors, including a tryptophan residue, two units of acetic acid, and an isoprenoid moiety with two intermediates (cyclo-acetoacetyl-L- tryptophan (cAATrp) (**211**) and *β*-cyclopiazonic acid (*β*-CPA) (**212**)) in the PKS–NRPS hybrid pathway [165]. Additionally, *α*-CPA was demonstrated to be a potent, selective, and reversible SERCA (sarco/endoplasmic reticulum Ca^2+^-ATPase) inhibitor in different tissues and cell types [166], and was observed to have an immunosuppressive effect [167] and antiviral activities against the Sendai virus, hepatitis B virus, rotavirus, and human respiratory syncytial virus via different mechanisms [168]. Since *α*-CPA was first isolated in 1968, 26 CPA-type tetramate alkaloids (**210**–**235** in Figure 9) have been reported from marine fungi *Aspergilli, Penicillium, Pseudallescheria*, and actinomycete *Amycolatopsis*. These marine CPA-type tetramic acids, containing the tetramic acid moiety as a critical structural motif, were characterized to have some structural variations, and all belong to the indole or oxindole (indolinone) subclasses of CPA-type tetramate alkaloids [165,166]. 

In 2009, iso-*α*-cyclopiazonic acid (**213**), along with its isomer **210**, was structurally characterized in the marine algae-derived *A. flavus* C-F-3 [169], the marine prawn-derived *A. flavus* OUCMDZ-2205 [170], and marine-derived *P. camemberti* [171]. Two new CPA derivatives, amycocyclopiazonic acid (**214**) and amycolactam (**215**), were isolated from a sponge-associated rare actinomycete *Amycolatopsis* sp. [172]. Very recently, a new CPA derivative, pseuboydone E (**216**), was isolated from the marine soft coral-derived *Pseudallescheria boydii* F19-1 [173]. Compounds **211** and **212** were confirmed to be the biosynthesized intermediates of CPA in the marine fungus *Aspergillus oryzae* HMP-F28 using biosynthetic machinery [174].

Metabolites **210**–**216** are the CPA derivatives belonging to the indole subclass of CPA-type tetramate alkaloids. All the remaining analogues, cyclopiamides (**217**–**226**), speradines (**227**–**235**), and aspergillines (**225**), were categorized in the oxindole subclass of CPA-type tetramate alkaloids [166]. The first *N*-methylated pentacyclic oxindole analogues of *α*-CPA, speradine A and 3-OH-speradine A (**227** and **228**), were isolated in cultures of the marine-derived fungi *A. tamarii* M143 and *A. oryzae* HMP-F28 [174,175,176]. Four other tetracyclic oxindole alkaloids, named speradine B, C, D, and E (**224**, **229**, **230,** and **231**), were identified from marine-sediment-derived *A. oryzae* [177]. A rare hexacyclic oxindole alkaloid, speradine F (also termed penicamedine A [171]) (**232**), together with two novel tetracyclic oxindoles, speradine G and H (**224** and **234**), were isolated from marine-sediment-derived *Aspergillus oryzae* [178]. The terminology for oxindoles has been incorrectly used in the literature. For example, Ma et al. [179] reported the identification of speradine B, C, and D from a sponge-derived strain of *A. flavus* MXH-X104, and Wang et al. [180] reported the identification of speradine B from a mangrove-derived strain of *P. dipodomyicola* Y26-02. However, these molecules do not correspond with the metabolites previously described in Hu’s report [177]. Another group of CPA-related oxindoles, cyclopiamides B–J (**218**–**226**), along with cyclopiamides **210**, **232**, and **234,** were isolated from a deep-sea-derived strain of *P. commune* DFFSCS026 [181]. Cyclopiamides H and I, separated in *P. commune*, were proven to be the same chemical entities as speradine B (**224**) and aspergilline D (isolated from the *A. versicolor* in 2014 [182]) (**225**), respectively. To avoid future confusion, we suggest renaming these compounds according to the chronology of their discovery, as follows: speradine B, C, and D [179], should be re-designated as speradine F, C’, and C (**232**, **235**, and **229**), speradine B in [180] should be renamed as 2-demethylsperadine F (**233**) [183], and speradine G in [178] as speradine B (**225**). Notably, **234** [179] possesses an unusual 6/5/6/5/5/6 hexacyclic system with a unique 4-oxo-1,3-oxazinane ring, and **225** [182], **226** [181], **232** [171], and **233** [180] bear an unusual rigid and sterically congested hexacyclic 6/5/6/5/5/5 indole-tetrahydrofuran-tetramate scaffold. 

The bioassay results showed that **210** has potent toxicity toward brine shrimp (IC_50_ < 1.0 μg/mL) [181] and cytotoxicity against four HTCLs (IC_50_ = 2.4–21.5 μM) [169] as well as antibacterial activity against E. coli [184]. However, its isomer (**213**) only displayed weak cytotoxicity against A549 (IC_50_ = 42.2 μM) [169]. Compound **215** displayed significant cytotoxicity towards the SNU638 and HCT116 cell lines (IC_50_ = 0.8, 2.0 μM), and moderate cytotoxicity against the A546, K562, and SK-HEP1 cell lines (IC_50_ = 13.7, 9.6, 8.3 μM) [172]. Compound **227** revealed inhibitory activity against SERCA (IC_50_= 8 μM) and inhibitory activity towards histone deacetylase (IC_50_ = 100 μg/mL), as well as antibacterial activity against Micrococcus luteus (MIC = 16.7 μg/mL) [175]. Compound **235** [179] displayed potent inhibition activity against Sf9 insect cells with IC_50_ = 0.9 μM. Speradine B (**225**) [182] displayed potent anti-TMV (tobacco mosaic virus) activity on nicotine tobacco leaf (IC_50_ = 38.9 μM, with the positive control of ningnamycin 30.5μM), protecting the host plant against TMV infection and presenting moderate cytotoxicity against five HTCLs (IC_50_ = 1.2–4.2 μM). Cyclopiamides (**217**–**226**) showed weak toxicity to brine shrimp (IC_50_ = 14.1–46.5 μg/mL) and displayed no cytotoxic (HepG-2 and HeLa) or antiviral (N1H1) activities [181].

### 2.8. Other Tetramic Acids

**Figure 10:**
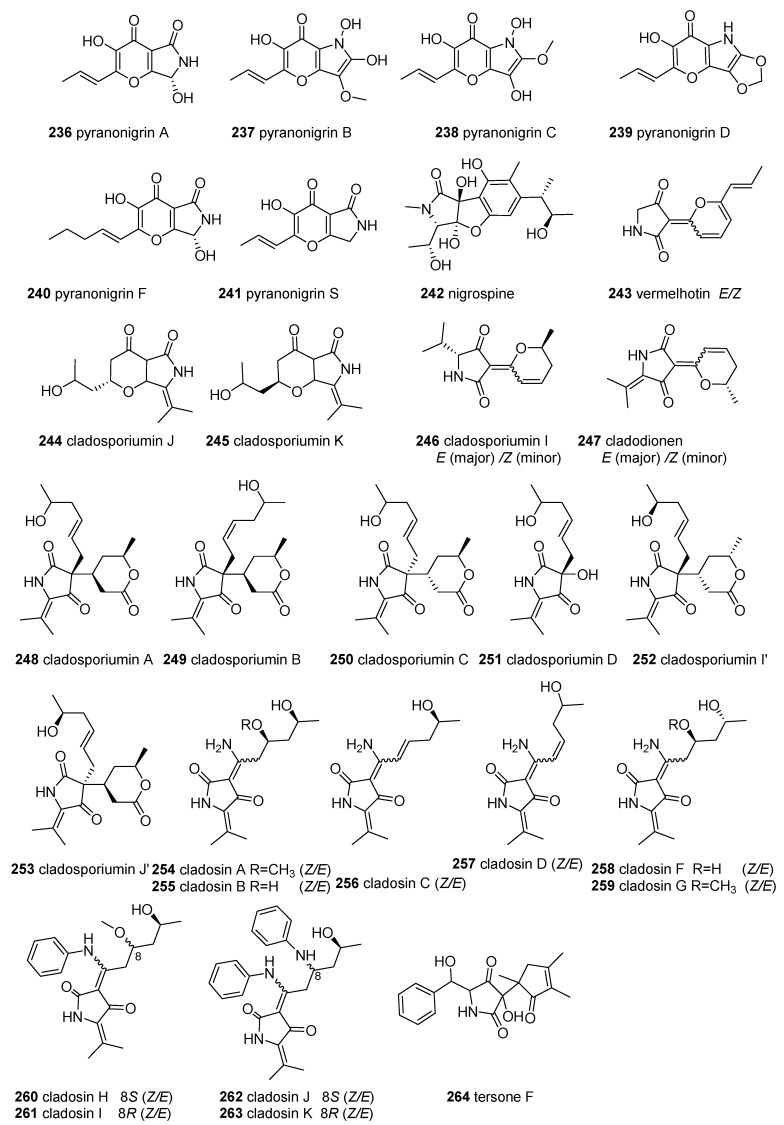

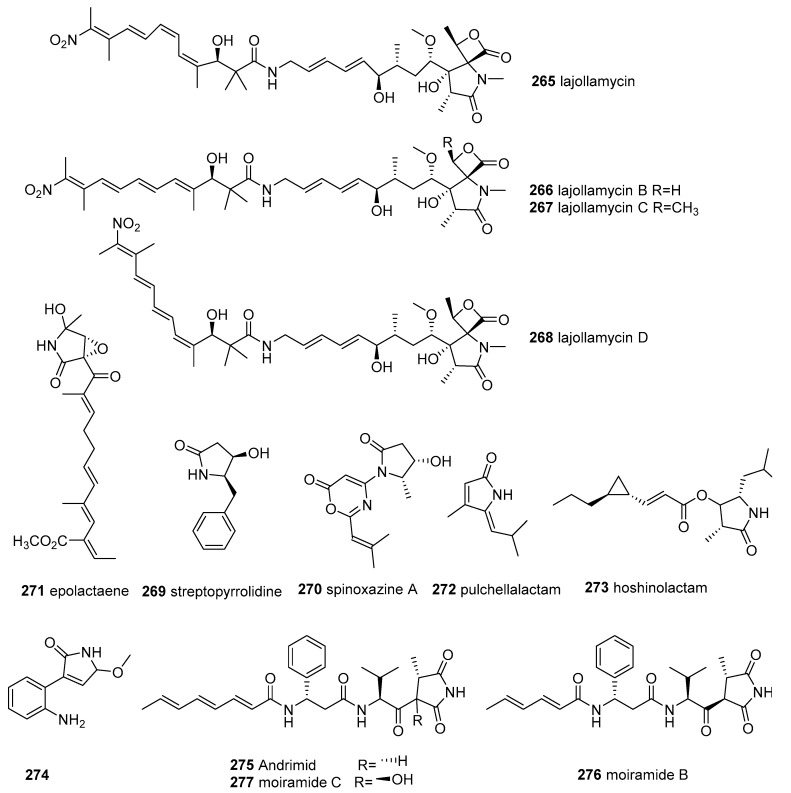
Chemical structures of other tetramic acids (**236**–**277**).

All of the smaller subgroups of TAs, whose numbers were less than 13, were called “other tetramic acids”, which include 42 compounds (**236**–**277** in Figure 10).

Pyranonigrins, featuring an unprecedented pyrano[3,2-b]pyrrole skeleton, were mainly isolated from the fungi *Aspergillus* and *Penicillium*. Pyranonigrin A (**236**) and pyranonigrins B–D (**237–239**) were initially obtained from the sponge-derived fungus *Aspergillu niger* Van Tieghem [185]. Subsequently, pyranonigrin A (**236**) and pyranonigrin S (**240**) were also purified from the extracts of the marine fungus *Aspergillus niger* LL-LV3020, and its original structure was revised as **236 [186]**. Pyranonigrin F (**241**) together with **236** were identified from the mangrove-derived *Penicillium brocae* MA-231 [187]. Interestingly, **236** was found to have extensive bioactivities, such as inhibition of the growth of neonate larval of the plant-insect *Spodoptera littoralis* [185] and suppression of the expression of vascular cell adhesion molecules (VCAM)-1 in human umbilical vein endothelial cells (HUVECs) induced by tumor necrosis factor (TNF)-*α* without affecting the cell viability of HUVECs [188], as well as displaying Epstein–Barr virus early antigen inhibitory activity [189]. Further, compounds **236** and **241** showed significant antimicrobial activities against human pathogens (*Staphyloccocus aureus*), aquatic bacteria (*Vibrio harveyi* and *V. parahaemolyticus*), and plant pathogens (*Alternaria brassicae* and *Colletotrichum gloeosprioides*) with an MIC of 0.5 μg/mL, which is more potent than the positive control chloromycetin and bleomycin (for plant pathogens) [187]. Furthermore, compound **240** exhibited a higher level of 1,1-diphenyl-2-picryhydrozyl (DPPH) radical scavenging activity than **236 [188,190,191]**. Nigrospine (**242**) with a rare 2,3-dihydro-benzofuran[2,3-c]2-pyrrolidone skeleton was purified from the marine-derived fungus *Nigrospora oryzae* SCSGAF 0111 [192].

Vermelhotin (**243**) was the first example of a TA with a C-3-pyrane ring. It was initially isolated as the E-isomer from the terrestrial fungus IFM52672 [193] and then obtained as an *E/Z* mixture from a marine sponge-associated unidentified fungus CRI247-01 [194]. Compound **243** displayed a full range of biological activities, such as potent inhibition of calmodulin by binding to calmodulin at site I [195], significant cytotoxicity against eleven HTCLs (IC_50_ = 0.31–13.5 μg/mL) [194], moderate antiplasmodial activity (IC_50_ = 1–10 μM) [194], anti-inflammatory activity through the inhibition of NO production (IC_50_ = 5.35 μM) in LPS-induced RAW 264.7 cells via inhibition of iNOS expression and p38 phosphorylation [196], and inhibition of the MDR-TB isolates *Mycobacterium* tuberculosis (MIC 1.5–12.5 μg/mL) [197].

Recently, deep-sea-sediment-derived fungi have been demonstrated to be the source of the chemical diversity of TAs. The genera of *Cladosporium* from deep-sea sediments are sources of many subclasses of tetramic acid derivatives with different C-3 substituent groups, including C3-acyl-linear side chains (simple 3-ATAs, cladosporiumins E-H and their Na complexes, cladosporiumins N-O, and L [31])), C3-dienoyl (cladosporiumin M [32]), pyrano[3,2-b] pyrone (cladosporiumins J and K [32]) (**244** and **245**), C3-pyrane ring (cladosporiumin I, and cladodionen [32]) (**246** and **247**), C3- linear side chains and pyran rings (cladosporiumins A-C [31], I’ and J’ [91]) (**248**–**250**, **252,** and **253**) and its tetramate precursor (cladosporiumin D [31]) (**251**), and C-3-imine-TA (cladosins A-K) (**254**–**263**). Notably, some of these compounds have special structures. For example, **248**–**250**, **252,** and **253** have a quaternary (C-3) center carrying a *trans*-hexylenic alcohol side chain and a six-membered lactone ring [31,91]. The pharmacological results showed that only compound **247** had cytotoxic activities against four HTCLs (MCF-7, HeLa, HCT-116, and HL-60), with IC_50_ values of 18.7, 19.1, 17.9, and 9.1 µM [198]. However, cladosporiumins I’ - J’ (**252**–**253**), and other cladosporiumins displayed weak or no cytotoxicity against the four breast HTCLs [31,91].

Ten cladosins (A–D, and F–K) (**254**–**263**), a series of C-3-imine-TA analogues, were found in deep-sea-derived *C. sphaerospermum.* Cladosins **254**–**257** were discovered from deep-sea-derived *C. sphaerospermum* 2005-01-E3 cultured with a rice-based medium [199]. Following the OSMAC method, cladosins F–G (**258**–**259**) were isolated from the soybean-based medium fermentation of this strain [200]. Using a SAHA (suberanilohydroxamic acid)-based epigenetic modification strategy, cladosins H–K (**260**–**263**), bearing an aniline–tetramic acid moiety, and a related compound, cladodionen, were obtained from the deep-sea-derived fungus, *C. sphaerospermum* L3P3, by the same group [201]. All of the cladosins existed as one pair of tautomeric mixtures, differing in their enamine configurations. Among them, **254**–**256** and **258**–**262** were isolated as an inseparable equilibrium mixture of two geometric isomers, exo-form A (Δ^3(6)^: *E*) and exo-form B (Δ^3(6)^: *Z*), with a ratio of 5:3, but **257** was opposite to the ratio of the tautomers [199,200,201]. Among the bioactivities of the cladosins, only **256** displayed mild anti-influenza A H1N1 virus activity with **254**–**259** [199,200], but the cladosins with an aniline moiety (**260**–**263**) showed enhanced cytotoxicity, especially **261,** which presented promising cytotoxicity against the HL-60 cell line (IC_50_ = 2.8 μM) [201]. The deep-sea fungus *Phomopsis tersa* produced tersone F (**264**), which was devoid of cytotoxic activities [202].

Lajollamycins, featuring a unique scaffold consisting of a 5-spiro-*β*-lactone-*γ*-lactam ring and a nitro-tetraene group, were mainly isolated from a *Streptomyces* strain. The culture fermentation of *Streptomyces nodosus* (NPS007994) from marine sediment yielded lajollamycin (**265**), which showed antimicrobial activity against *E.coli* and three pairs of drug-sensitive and -resistant Gram-positive bacteria (MIC= 1.5–20 μg/mL) and inhibited the growth of B16-F10 tumor cells (EC_50_ = 9.6 μm) [203]. Another marine-derived *Streptomyces* sp., SMC72, isolated from a seashore sediment sample, produced a series of lajollamycin derivatives, including lajollamycins B–D (**266**–**268**) and lajollamycin (**265**), which displayed moderate inhibitory activities against *Candida albicans* isocitrate lyase (ICL) [204].

Streptopyrrolidine (**270**) was sourced as a metabolite of the marine-derived *Streptomyces* sp. KORDI-3973 and displayed significant anti-angiogenesis activity [205]. Spinoxazine A (**270**), with a *γ*-lactam moiety and a 1,3-oxazin-6-one system, was obtained from marine-derived *Streptomyces spinoverrucosus* collected in the Bahamas and lacked bioactivity against four HTCLs and two bacterial strains [206].

Epolactaene (**271**), with a long-chain-substituted *γ*-lactam group, was discovered from the marine fungus *Penicillium* sp. BM 1689-P and displayed neuritogenic properties by arresting the cell cycle at the G0/G1 phase and inducing the outgrowth of neurites in human neuroblastoma SH-SY5Y cells [207], selectively inhibiting the activities of mammalian DNA polymerases *α* and *β* as well as human DNA topoisomerase II [208], and could combine with Hsp60 as a Michael acceptor to inhibit Hsp60 chaperone activity [209,210]. Pulchellalactam (**272**) was reported from the marine-derived-fungus *Corollospora pulchella* and was used as a selective inhibitor of the CD45 protein, tyrosine phosphatase [211].

When screened for antiprotozoal activity from a marine cyanobacterium, *Oscillatoria* sp. yielded hoshinolactam (**273**), which showed potent antitrypanosomal activity against *Trypanosoma brucei brucei* GUT, with an IC_50_ value of 3.9 nM (equivalent to the positive control pentamidine: IC_50_ = 4.7 nM) and no cytotoxicity against MRC-5 cells (IC_50_ > 25 μM) [212].

The 3-(2-amino-phenyl)-5-methoxy-1,5-dihydro-pyrrol-2-one (**274**) was identified as a metabolite of a novel marine bacterium *Rapidithrix thailandica* and displayed moderate but selective antibacterial activity against *VRE* [213].

Andrimid (**275**), moiramides B-C (**276**–**277**), and their precursor, moiramide A, were produced by the bacterium *Pseudomonas fluorescens*, isolated from marine tunicates [214]. In contrast to moiramide A and **277**, both **275** and **276** showed antibacterial activity, highlighting that the intact succinimide moiety is the critical pharmacophore [214]. Compound **275** was active in inhibiting *MRSA* (MIC: 2 µg/mL) and *VRE* (32 µg/mL), while **276**, the congener with the shortest polyene chain, was more potent in its inhibition against both *MRSA* (0.5 µg/mL) and *VRE* (4 µg/mL) [214]. Further studies revealed that **275** and **276** had broad-spectrum antibacterial activity as a class of a new potent bacterial acetyl-CoA carboxylase inhibitor, targeting its fatty acid biosynthesis [215] and highlighting the fatty acid side chain and the pyrrolidinedione moiety as the most important pharmacophores [216].

## 3. Summary of Tetramic Acid Products from Marine Microbes

**Figure 11:**
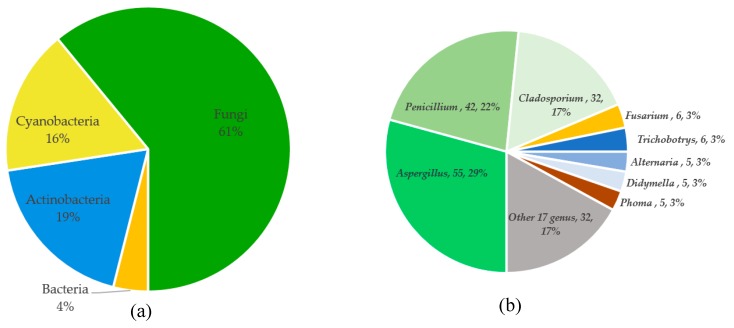
(**a**) The tetramic acids (TAs) from marine microorganisms in this review divided by the origin of microorganisms, indicating that fungi are the dominant source. (**b**) The pie chart provides more in-depth insight into the fungi.

**Figure 12:**
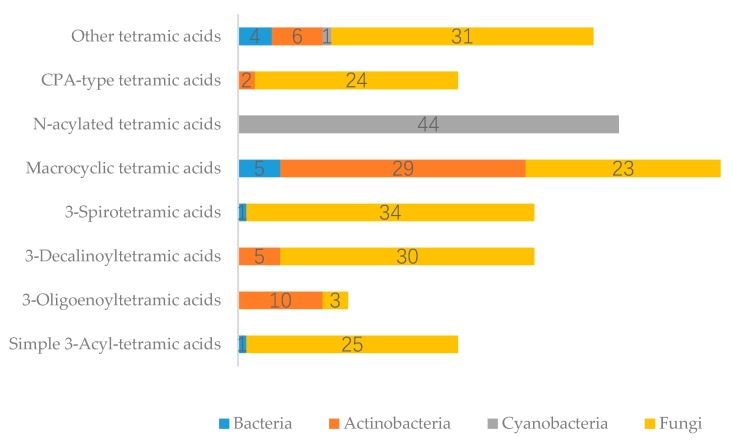
The relationship between different chemical groups of TAs and their producer (marine microorganisms). This number corresponds to the number of TA compounds in different chemical classes.

TAs were sourced from a diverse range of marine microorganisms. Culturable marine fungi provided the majority of natural TAs and belonged to 25 genera (*Aspergillus, Penicillium, Cladosporium, Fusarium, Trichobotrys, Alternaria, Didymella, Phoma, Chaunopycnis, Cochliobolus, Tolypocladium, Ascochyta, Xylariaceae, Lindgomycetaceae, Zopfiella, Beauveria, Corollospora, Epicoccum, Nigrospora, Phomopsis, Pleosporale, Pseudallescheria, Trichoderma, Westerdykella, Microdiplodia*, and unknown fungi). Other sources were from Actinobacteria (four genera, *Streptomyces, Actinoalloteichus, Amycolatopsis, Micromonospora*,), Cyanobacteria (seven families, *Moorea producens, Oscillatoria, Symploca, Caldora, Schizothrix, Symploca, Okeania*, and unidentified assemblages), and Bacteria (six genera, *Rapidithrix, Pseudomonas, Vibrio, Bacillus, Alteromonas*, and *Pseudoalteromonas).* Fungi were the dominant producers of the 277 TAs, with 61% of marine microbe-derived TAs from fungi (25 fungal genera), 19% from Actinobacteria (86.5% from the genus of *Streptomyces*), 16% from Cyanobacteria (mainly from *Moorea producens*, formerly named as *Lyngbya majuscula*), and only 4% from Bacteria (Figure 11a), with marine fungi consistently observed to be the dominant source of MNPs in the last ten years [10,11]. Within the individual fungi groups, the *Aspergillus* (29%)*, Penicillium* (22%)**, and *Cladosporium* (17%) species were the predominant fungal sources of TAs (Figure 11b). Based on an analysis of the relationship between the different chemical groups of TAs and their producers (Figure 11), it can be concluded that the positive correlation between the structures of metabolites and their producer microbes is related to their special BGCs. As seen in Figure 12, fungi can produce significant classes of compounds, including simple 3-ATA (96% of this class of compounds, mainly from *Penicillium, Cladosporium*, and *Aspergillus*), 3-STA (97%, mostly from *Aspergillus*), CPA-type TAs (92%, mostly from *Aspergillus* and *Penicillium*), 3-DTA (86%, from 12 species), others (74%, mainly from *Cladosporium* and *Aspergillus*), MTAs (40%, all PTAs, mainly from *Penicillium*, and *Didymella*), and 3-OTA (23%, from *Aspergillus* and *Cladosporium*). All of the *N*-acylated TAs were produced by cyanobacteria (mostly from *Moorea producens*) as their marker compounds. Actinobacteria (mostly *Streptomyces*) can provide five groups of TAs: 3-OTA (77%, from *Streptomyces)*, MTAs (51%, mainly PTMs, mainly from *Streptomyces*), 3-DTA (14%, mainly from *Streptomyces*), others (14%, all from *Streptomyces*), and 3-STA (3%, from *Amycolatopsis*).

**Figure 13:**
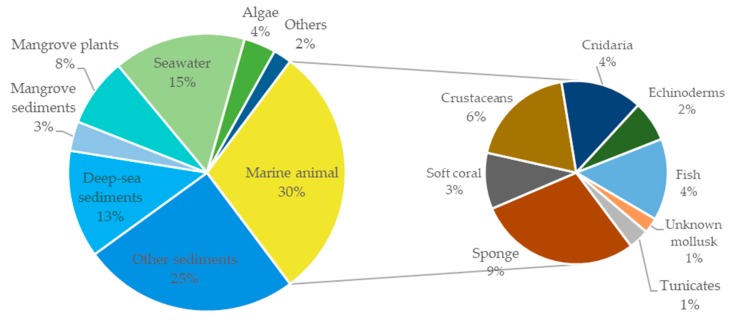
The TAs from marine microorganisms were divided by their sources (habitats); 277 TAs were isolated from 120 species of microorganisms in 120 habitats.

When looking at the habitats/sources of these marine microorganisms for TAs in Figure 13, 56% of the compounds were isolated from marine environments (i.e., marine sediments (41%), and seawater (15%) (mainly cyanobacteria)), while the remaining compounds were obtained from living matter, i.e., marine animals (30%) and aquatic plants (12%). Within the individual groups, other marine sediments (25%), seawater (15%), deep-sea sediments (13%), mangrove habitats (11%), sponges (9%), and crustaceans (6%) were the most predominant sources of microorganisms. A newly emerging source is the extreme environment, i.e., deep-sea sediments (13%), which can produce structurally unique metabolites.

**Figure 14:**
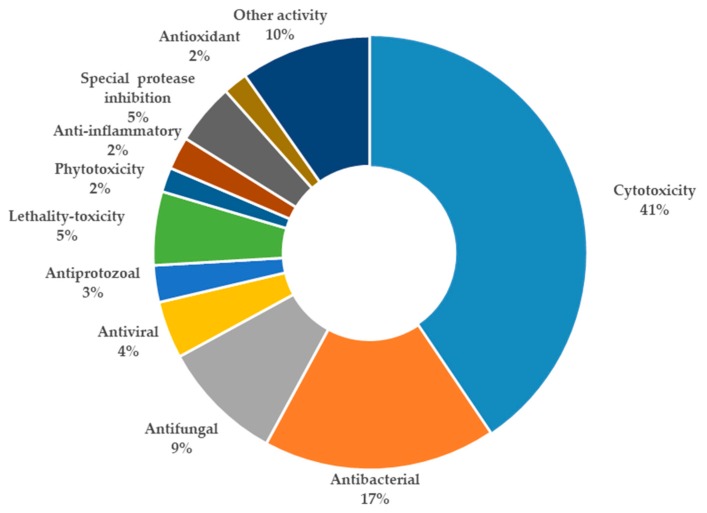
The percentage represents the proportion of one activity compared to the whole occurrence of activities of 202 bioactive TAs from marine microorganisms (*n* = 327). Some compounds present various activities and are counted in more than one category.

In the bioassay of the 261 tetramic acids (94.2% compounds) from marine microorganisms, 77.4% of compounds (202) displayed various activities (*n* = 327) and, on average, exhibited 1.62 activities per bioactive-TA. This result is because some compounds presented various activities and were counted in more than one category. The ten major bioactivities are listed in Figure 14 (cytotoxicity, antibacterial, antifungal, antiviral, antiprotozoal, lethality–toxicity, phytotoxicity, anti-inflammatory, and antioxidant activities, as well as special protease enzyme inhibition activities). Cytotoxicity (40%) was the most significant pharmacological activity, with up to 132 compounds among the 327 listed compounds, which inhibited the proliferation of different tumor cell lines in vitro, followed by anti-infective/antimicrobial activities (30%), including antibacterial activities for 57 compounds (17%), antifungal activities for 30 compounds (9%), and antiviral activities for 14 compounds (4%). This result is consistent with the focus of medical research, as tumors and infectious diseases remain the primary threat to human health in modern society. Other selected major activities included lethality–toxicity for 18 compounds (5%), special protease inhibition for 15 compounds (5%), and antiprotozoal activity for 10 compounds (3%).

**Figure 15:**
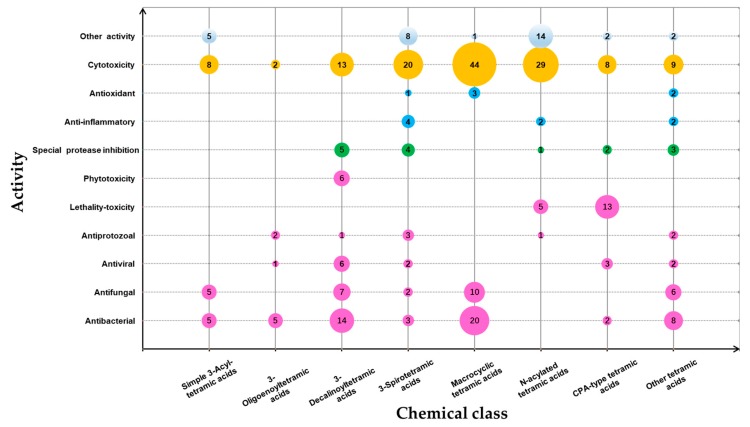
Classification of the 202 bioactive TAs according to their activities and chemical classes. The number of compounds is symbolized by the disc diameters for each bioactivity and each chemical class. The colors correspond to the different categories of the activity targets. Gray represents a mixed target; yellow mainly represents a cell line target, blue primarily represents the specific cellular mechanism, green represents the enzyme target, and purple represents the entire organism target.

The number of different chemical classes of TAs displaying each bioactivity is shown in Figure 15. The bioactivities of the compounds were evaluated for different targets, ranging from a specific cellular mechanism to the entire organism. For example, the inhibitory activity of special protease was shown to target enzymatic processes when antiprotozoal, lethality–toxicity, phytotoxicity, and antimicrobial activity were tested against whole organisms. Further, cytotoxicity was based on the cell line level, and some research is related to their specific cellular and molecular mechanisms; anti-inflammatory and antioxidant activities are mainly assessed on the basis of specific cellular mechanisms, which may also be included in cytotoxicity and other activities. The present analysis confirms the preceding observations (i.e., that cytotoxicity is the most common bioactivity, followed by antibacterial and antifungal activity). Some activities were displayed only for certain compounds: phytotoxicity involved only 3-D TAs; lethality–toxicity involved only *N*-acylated TAs and CPA-type TAs; anti-inflammatory activity was observed for 3-STAs, *N*-acylated TAs, and other TAs; and antioxidant activity was observed for 3-STAs, MTAs, and other TAs. For the chemical classes, no specific activities were observed for one chemical class concerning different types. Four chemical classes (3-STAs, 3-DTAs, *N*-acylated TAs, and MTAs) seem to present a relatively more extensive set of activities. 

## 4. Conclusions and Outlooks 

This review has provided a comprehensive overview of 277 tetramic acid products from 120 marine-derived microbes (containing fungi, actinobacteria, bacteria, and cyanobacteria), presented by their structural characteristics and covering up to September 2019, with 195 research publications related to tetramic acids and their bioactivities. Marine fungi are the dominant source of the rapidly increasing numbers of TAs, of which the *Aspergillus, Penicillium, Cladosporium* species are the predominant marine microbe sources of TAs. Most TAs (77.4%) displayed various pharmacological activities, especially cytotoxicity (40%). Interestingly, deep-sea sediment-derived fungi have become an essential source of the unique structure of bioactive tetramic acids. 

As microbial-derived compounds will almost certainly dominate the MNP field in the coming sesquidecade [11], the tetramic acid compounds from marine-derived microorganisms will reveal increasingly greater biological and chemical diversity. Because of the relative ease of collecting marine microbes, a wide variety of approaches for natural product discovery (including metagenomics and genome mining approaches, the heterologous expression method, the OSMAC approach, and chemical epigenetic modification) can be used, as well as advanced and combinational methods for metabolite identification, and several public, private, and commercial databases for rapid dereplication. The various pharmacological properties displayed by TAs with intriguing structures provide medicinal chemists with a variety of potential lead compounds for the development of marine drugs. 

## Supplementary Materials

The following are available online at https://www.mdpi.com/1660-3397/18/2/114/s1, Table S1: The tetramic acid compounds from marine-derived microorganisms.
