# Supplementary material for: The Biological and Chemical Diversity of Tetramic Acid Compounds from Marine-Derived Microorganisms
Source: Mar Drugs. 2020 Feb 15;18(2):114. doi: 10.3390/md18020114 (PMC7074263; doi:10.3390/md18020114)
Supplement: Supplementary file 1 [file marinedrugs-18-00114-s001.pdf]

The Biological and Chemical Diversity of Tetramic Acid Compounds from Marine-Derived Microorganisms

Minghua Jiang [1,2,†], Senhua Chen [1,2,3,†,*], Jing Li [1,2,3] and Lan Liu [1,2,3,*]

[1] School of Marine Sciences, Sun Yat-sen University, Guangzhou 510006, China; jiangmh23@mail2.sysu.edu.cn; chensenh@mail.sysu.edu.cn; lijing356@mail.sysu.edu.cn cesllan@mail.sysu.edu.cn;
[2] South China Sea Bio-Resource Exploitation and Utilization Collaborative Innovation Center, Guangzhou 510006, China;
[3] Southern Laboratory of Ocean Science and Engineering (Guangdong, Zhuhai), Zhuhai 519000, China;
* Correspondence: chensenh@mail.sysu.edu.cn.; cesllan@mail.sysu.edu.cn; Tel.: +86-020-84725459 (S.C. & L.L.)
† These authors contributed equally to this work.

Table S1: The Tetramic Acid Compounds from Marine-Derived Microorganisms

Compound Name (NO.)	Isolate marine microorganisms	microorganisms category	Host/Origin and Location	Biological Activity	References
Magnesidin A (**1**)	*Vibrio gazogenes* ATCC29988	Bacteria	marine mud, unknown place		17
	Pseudomonas magnesiorubra nov. sp. ATCC 21856	Bacteria	Marine alga, *Caulerpa peltata*, Bombay, India	antibacterial	18
Epicoccamide (**2**)	*Epicoccum purpurascens*	Fungal	jellyfish *Aurelia aurita*, North Sea, Germany	NF	19
Penicillenol A1 (**3**)	*Penicillium* sp. GQ-7	Fungal	mangrove *Aegiceras corniculatum*, Gaoqiao, China	cytotox. vs. HL-60, A-549, BEL-7402, P388 (IC_{50} =0.76, 23.8, 13.03, 8.85μM)	20
	Penicillium citrinum		sediment, Min River, China	mod. cytotox. vs. A375 (IC_{50} =12.8 μM)	24
	Xylariaceae sp. SCSGAF0086		gorgonian coral *Melitodes squamata*, South China Sea	anti-TB, 96.1% inhibition 10μM	25
	Aspergillus restrictus DFFSCS006		marine sediment, South China Sea	inhibit biofilm formation of *Candida albicans*, inhibitory rate 38% at 50 μg/mL	26
Penicillenol A2 (**4**)	*Penicillium* sp. GQ-7	Fungal	mangrove *Aegiceras corniculatum*, Gaoqiao, China	cytotox. vs. HL-60 (IC_{50}=16.26 μM)	20
	Penicillium citrinum		sediment, Min River, China	weak cytotox. vs. A375 (IC_{50} = 44.6 μM)	24
	Xylariaceae sp. SCSGAF0086		gorgonian coral *Melitodes squamata*, South China Sea	-	25

	Aspergillus restrictus DFFSCS006		marine sediment, South China Sea	inhibit biofilm formation of *Candida albicans*, inhibitory rate 81% at 50 μg/mL	26
Penicillenol B1 (**5**)	*Penicillium* sp. GQ-7	Fungal	mangrove *Aegiceras corniculatum*, Gaoqiao, China	cytotox. vs. HL-60 (IC_{50} =3.20μM)	20
	Penicillium citrinum		sediment, Min River, China	mod. cytotox. vs A375, (IC_{50} =10 μM)	24
	Aspergillus restrictus DFFSCS006		marine sediment, South China Sea	inhibit biofilm formation of *Candida albicans*, inhibitory rate 75% at 50 μg/mL	26
				antibacterial vs. *Staphylococcus aureus*, abs. config. Assigned	23
Penicillenol B2 (**6**)	*Penicillium* sp. GQ-7	Fungal	mangrove *Aegiceras corniculatum*, Gaoqiao, China	cytotox. vs. HL-60 (IC_{50} =7.65 μM)	20
	Penicillium citrinum		sediment, Min River, China	mod. cytotox. vs A375 (IC_{50} = 3.46 μM)	24
	Aspergillus restrictus DFFSCS006		marine sediment, South China Sea	inhibit biofilm formation of *Candida albicans*, inhibitory rate 54% at 50 μg/mL	26
				antibacterial vs. *Staphylococcus aureus*, abs. config. Assigned	23
Penicillenol C1 (**7**)	*Penicillium* sp. GQ-7	Fungal	mangrove *Aegiceras corniculatum*, Gaoqiao, China	no cytotox.	20
	Aspergillus restrictus DFFSCS006		marine sediment, South China Sea	–	26
Penicillenol C2 (**8**)	*Penicillium* sp. GQ-7	Fungal	mangrove *Aegiceras corniculatum*, Gaoqiao, China	no cytotox.	20
	Aspergillus restrictus DFFSCS006		marine sediment, South China Sea	inhibit biofilm formation of *Candida albicans*, inhibitory rate 58% at 50 μg/mL	26
Penicillenol D (**9**)	*Trichoderma citrinoviride*	Fungal	marine sediments, Langqi Island, Fujian, China	cytotox. vs. A-375 (IC_{50} =32.6μM)	21
Penicillenol D1 (**10**)	*Penicillium citrinum*	Fungal	marine sediments, Langqi Island, Fujian, China	weak cytotox. vs. A-549, HL-60	22
Penicillenol D2 (**11**)	*Penicillium citrinum*	Fungal	marine sediments, Langqi Island, Fujian, China	weak cytotox. vs. A-549, HL-60	22
Penicitrinine A (**12**)	*Penicillium citrinum*	Fungal	marine sediments, Langqi Island, Fujian, China	stronger cytotox. than p.c., via cell apoptosis	27

Chaunolidine A (**13**)	*Chaunopycnis* sp. (CMB-MF028)	Fungal	marine *Siphonaria* sp., Queensland, Australia	metal chelation capacity	28
Chaunolidine B (**14**)	*Chaunopycnis* sp. (CMB-MF028)	Fungal	marine *Siphonaria* sp., Queensland, Australia	metal chelation capacity	28
Chaunolidine C (**15**)	*Chaunopycnis* sp. (CMB-MF028)	Fungal	marine *Siphonaria* sp., Queensland, Australia	modest Gram-positive antibacterial (IC_{50} = 5– 10 μM), metal chelation capacity	28
F-14329 (**16**)	*Chaunopycnis* sp. (CMB-MF028)	Fungal	marine *Siphonaria* sp., Queensland, Australia	antilipidemic effect, metal chelation capacity	28, 29
Tolypocladenol A1 (**17**)	*Tolypocladium geodes* sp. MF458	Fungal	marine sponge, unknown place	NF	30
Tolypocladenol A2 (**18**)	*Tolypocladium geodes* sp. MF458	Fungal	marine sponge, unknown place	NF	30
Tolypocladenol C (**19**)	*Tolypocladium geodes* sp. MF458	Fungal	marine sponge, unknown place	NF	30
Cladosporiumin E (**20**)	*Cladosporium* sp. SCSIO z0025	Fungal	deep-sea sediment, Okinawa Trough, Japan	NF	31
Cladosporiumin F (**21**)	*Cladosporium* sp. SCSIO z0025	Fungal	deep-sea sediment, Okinawa Trough, Japan	NF	31
Cladosporiumin G (**22**)	*Cladosporium* sp. SCSIO z0025	Fungal	deep-sea sediment, Okinawa Trough, Japan	NF	31
Cladosporiumin H (**23**)	*Cladosporium* sp. SCSIO z0025	Fungal	deep-sea sediment, Okinawa Trough, Japan	NF	31
Cladosporiumin N (**24**)	*Cladosporium sphaerospermum* EIODSF 008	Fungal	deep-sea sediment, East Indian Ocean	NF	32
Cladosporiumin O (**25**)	*Cladosporium sphaerospermum* EIODSF 008	Fungal	deep-sea sediment, East Indian Ocean	NF	32
Cladosporiumin L (**26**)	*Cladosporium sphaerospermum* EIODSF 008	Fungal	deep-sea sediment, East Indian Ocean	NF	32
18-OH-RKB-3384A (**27**)	*Aspergillus* sp. OUCMDZ-1914	Fungal	Mangrove Soils, Wenchang Hainan, China	NF	33
RKB-3384A (**28**)	*Aspergillus* sp. OUCMDZ-1914	Fungal	Mangrove Soils, Wenchang Hainan, China	anti-virus (H1N1 influenza virus), (IC_{50}=116.2 μM).	33
Cladosporiumin M (**29**)	*Cladosporium sphaerospermum* EIODSF 008	Fungal	deep-sea sediment, East Indian Ocean	NF	32

Tirandamycin A (**30**)	*Streptomyces* sp. 307-9	Actinomycete	marine sediments, Virgin Islands, USA	–	38
	Streptomyces tirandamycinicus sp. nov.		Marine Sponge, Wenchang, Hainan, China	antibacterial vs. *Streptococcus agalactiae (*MIC=2.52 μg/mL)	39
	Streptomyces sp. SCSIO 1666		marine sediments, South China Sea	–	37
	Streptomyces sp. URI-F11		marine sediments, Fisher's Island Sound, NY, USA	antiamoebic, (EC_{50}=44.3–46.3μM)	41
	Streptomyces sp. SCSIO 41399		*Porites sp.* Coral, Wenchang, Hainan, China	potent antibacterial vs. *Streptococcus agalactiae* (MIC= 5.9 μM)	42
				antibacterial inhibits chain initiation and elongation of Bacterial RNA polymerase	34
Tirandamycin B (**31**)	*Streptomyces* sp. 307-9	Actinomycete	marine sediments, Virgin Islands, USA		38
	Streptomyces tirandamycinicus sp. nov.,		Marine Sponge, Wenchang, Hainan, China	antibacterial vs. *Streptococcus agalactiae (*MIC=2.55μg/mL)	39
	Streptomyces sp. SCSIO 1666		marine sediments, South China Sea		37
	Streptomyces sp. SCSIO 41399		*Porites sp.* Coral, Wenchang, Hainan, China	potent antibacterial effects vs. *Streptococcus agalactiae* (MIC= 5.7μM)	42
				inhibited the parasitic nematode *Brugia malayi* at 30 μM	40
Tirandamycin C (**32**)	*Streptomyces* sp. 307-9	Actinomycete	marine sediments, Virgin Islands, USA	antibacterial including Gram-negative and VRE, MRSEs, (MICs 0.78–25μM)	46
	Streptomyces sp. SCSIO 1666		marine sediments, South China Sea	–	37
Tirandamycin D (**33**)	*Streptomyces* sp. 307-9	Actinomycete	marine sediments, Virgin Islands, USA	antibacterial	46
	Streptomyces sp. SCSIO 1666		marine sediments, South China Sea	-	37
Isotirandamycin B (**34**)	*Streptomyces* sp. SCSIO 41399	Actinomycete	*Porites sp.* Coral, Wenchang, Hainan, China	potent antibacterial effects vs. *Streptococcus agalactiae* (MIC= 11.5μM)	42
Tirandamycin E (**35**)	*Streptomyces* sp. SCSIO 1666	Actinomycete	marine sediments, South China Sea	-	43

Tirandamycin F (**36**)	*Streptomyces* sp. SCSIO 1666	Actinomycete	marine sediments, South China Sea	-	43
Tirandamycin C2 (**37**)	*Streptomyces* sp. SCSIO 1666	Actinomycete	marine sediments, South China Sea	-	44
Pre-tirandamycin (**38**)	*Streptomyces* sp. SCSIO 1666	Actinomycete	marine sediments, South China Sea	-	45
Tirandamycin K (**39**)	*Streptomyces* sp. 307-9	Actinomycete	marine sediments, Virgin Islands, USA	NF	46
Equisetin (**40**)	*Fusarium equiseti* D39	Fungal	unidentified mangrove plant, Yellow Sea, Qingdao, China	remarkable anti-phytopathogenic Bacterial and Fungal activities superior to the p.c., obvious phytotoxicity	47
	Fusarium sp. 152		deep-sea sediment, South China Sea	anti-*MRSA* (MIC = 1 μg/mL), antimicrobial, anti-HIV, cytotoxicity, phytotoxicity	48,7
epi-Equisetin (**41**)	*Fusarium equiseti* D39	Fungal	unidentified plant, Yellow Sea, Qingdao, China	remarkable anti-phytopathogenic Bacterial and Fungal activities superior to the p.c., obvious phytotoxicity	47
	Fusarium sp. 152		deep-sea sediment, South China Sea	–	48
Ascosalipyrrolidinone A (**42**)	*Ascochyta salicorniae*	Fungal	green alga *Ulva* sp., North Sea, To¨nning, Germany	antiplasmodial, antifungal, antibacterial, and inhibiting tyrosine kinase p56lck (anti-HIV).	49
Ascosalipyrrolidinone B (**43**)	*Ascochyta salicorniae*	Fungal	green alga *Ulva* sp., North Sea, To¨nning, Germany	-	49
Zopfiellamide A (**44**)	*zopfiella latipes* CBS 611.97.	Fungal	soil sample, Indian Ocean	mod. antibacterial, MIC 2-10μg/mL, antifungal, MIC=2μg/mL	50
Zopfiellamide B (**45**)	*zopfiella latipes* CBS 611.97.	Fungal	soil sample, Indian Ocean	very weak antibacterial, antifungal, MIC=2μg/mL	50
Sch210972 (**46**)	*Microdiplodia* sp.	Fungal	seaweed, North Sea, Germany	inhibits human leucocyte elastase (IC_{50}= 1.04 μg/mL), mod. antibacterial vs. B. megaterium	51
				Chemokine Receptor CCR-5 Inhibitor, IC_{50} = 79 nM	52
Beauversetin (**47**)	*Beauveria bassiana*	Fungal	sponge *Myxilla incrustans*, North Sea, Germany	mod. cytotox. of 6 HCLs, IC_{50} 3.09μg/mL	51
Trichobotrysin A (**48**)	*Trichobotrys effuse* DFFSCS021	Fungal	deep-sea sediment, South China Sea	cytotox. vs. KG-1a, IC_{50} =5.44μM, antiviral vs. HSV-1, IC_{50}= 3.08μM,	53
Trichobotrysin B (**49**)	*Trichobotrys effuse* DFFSCS021	Fungal	deep-sea sediment, South China Sea	cytotox. vs. KG-1a, IC_{50} =8.97μM, antiviral vs. HSV-1, IC_{50}= 9.37μM,	53
Trichobotrysin C (**50**)	*Trichobotrys effuse* DFFSCS021	Fungal	deep-sea sediment, South China Sea	cytotox. vs. KG-1a and antiviral t vs. HSV-1, IC_{50}<20μM	53

Trichobotrysin D (**51**)	*Trichobotrys effuse* DFFSCS021	Fungal	deep-sea sediment, South China Sea	cytotox. vs. KG-1a, IC_{50} = 6.16μM, mod. cytotox. 4 HCLs, IC_{50}<40 μM, antiviral vs. HSV-1, IC_{50}= 3.12μM,	53
Trichobotrysin E (**52**)	*Trichobotrys effuse* DFFSCS021	Fungal	deep-sea sediment, South China Sea	NF	53
Lindgomycin (**53**)	*Lindgomycetaceae* KF970	Fungal	the sponge of the *Kiel Fjord*, Baltic Sea, Germany	anti-Bacterial including *MRSA* and Fungal activities, IC_{50}= 2.2–17.8 μM	54
Ascosetin (**54**)	*Lindgomycetaceae* LF327	Fungal	the Antarctic	antibacterial including *MRSA* and Fungal activities, IC_{50}= 2.8–14.8 μM	54
				antibacterial (G+) including *MRSA* (MIC 2–16μg/mL), antibacterial (G-) haemophilus influenzae (MIC 8 μg/mL)	55
Iqalisetin A (**55**)	*Tolypocladium* sp.	Fungal	marine sediment, Frobisher Bay, Nunavut, Canada	NF	56
Iqalisetin B (**56**)	*Tolypocladium* sp.	Fungal	marine sediment, Frobisher Bay, Nunavut, Canada	NF	56
Lydicamycin (**57**)	*Streptomyces* platensis TP-A0598	Actinomycete	seawater sample, Toyama Bay, Japan,	antibacterial vs. Gram-positive Bacteria including *MRSA*, MIC 0.78-6.25μg/mL	57,68
TPU-0037-A (**58**)	*Streptomyces* platensis TP-A0598	Actinomycete	seawater sample, Toyama Bay, Japan,	antibacterial vs. Gram-positive Bacteria including *MRSA*, MIC 1.56-12.5μg/mL	57
TPU -0037-B (**59**)	*Streptomyces* platensis TP-A0598	Actinomycete	seawater sample, Toyama Bay, Japan,	antibacterial vs. Gram-positive Bacteria including *MRSA*, MIC 6.25-12.5μg/mL	57
TPU -0037-C (**60**)	*Streptomyces* platensis TP-A0598	Actinomycete	seawater sample, Toyama Bay, Japan,	antibacterial vs. Gram-positive Bacteria including *MRSA*, MIC 0.39-3.13μg/mL	57
TPU -0037-D (**61**)	*Streptomyces* platensis TP-A0598	Actinomycete	seawater sample, Toyama Bay, Japan,	antibacterial vs. Gram-positive Bacteria including *MRSA*, MIC 0.78-12.5μg/mL	57
Streptosetin A (**62**)	Actinomycete strain (CP13-10)	Actinomycete	marine sediment, San Francisco Bay, USA	weak inhibitory vs. yeast Sir2p and human SIRT1 and SIRT2.	59
Cladosporitin A (**63**)	*Cladosporium* sp. HNWSW-1	Fungal	mangrove root of *Ceriops tagal*, Hainan China	NF	60
Cladosporitin B (**64**)	*Cladosporium* sp. HNWSW-1	Fungal	mangrove root of *Ceriops tagal*, Hainan China	cytotox. vs. BEL-7042, K562, SGC-7901, IC_{50}=25–42.4 μM	60
Talaroconvolutin A (**65**)	*Cladosporium* sp. HNWSW-1	Fungal	mangrove root of *Ceriops tagal*, Hainan China	cytotox. vs. Hela, BEL-7042, IC_{50}=14.7–27.8 μM, inhibit α-glycosidase, IC_{50} =78.2 μM	60
Altercrasin A (**66**)	*Alternaria* sp. OUPS-117D-1	Fungal	sea urchin *Anthocidaris crassispina*, Osaka bay, Japan	Mod. cytotox. vs. P388, HL-60, L1210 (IC_{50}= 36.2, 21.5, 22.1μM)	61,62

Altercrasin B (**67**)	*Alternaria* sp. OUPS-117D-1	Fungal	sea urchin *Anthocidaris crassispina*, Osaka bay, Japan	cytotox. vs. P388, HL-60, L1210 (IC_{50}= 20, 12, 8μM)	62
Altercrasin C (**68**)	*Alternaria* sp. OUPS-117D-1	Fungal	sea urchin *Anthocidaris crassispina*, Osaka bay, Japan	mod. cytotox. vs. P388, HL-60, L1210 (IC_{50}=27–62μM)	62
Altercrasin D (**69**)	*Alternaria* sp. OUPS-117D-1	Fungal	sea urchin *Anthocidaris crassispina*, Osaka bay, Japan	cytotox. vs. L1210 (IC_{50}= 8.4μM) PC388–9.7μM, HL-60– 6.1μM equal to 5-fluorouracil	62
Altercrasin E (**70**)	*Alternaria* sp. OUPS-117D-1	Fungal	sea urchin *Anthocidaris crassispina*, Osaka bay, Japan	cytotox. vs. P388, L1210, HL-60 (IC_{50}= 15.5, 10.3, 6.2 μM)	62
Fusarisetin A (**71**)	*Fusarium equiseti* D39	Fungal	unidentified plant, Yellow Sea, Qingdao, China	prominent phytotoxicity, potent inhibition of metastasis in MDAMB-231, significant cytotox.	47
				acinar morphogenesis inhibitor, cancer migration inhibitor	63-65
Fusarisetin B (**72**)	*Fusarium equiseti* D39	Fungal	unidentified plant, Yellow Sea, Qingdao, China	potent phytotoxicity,	47
				cancer migration inhibitor	64,65
Fusarisetin C (**73**)	*Fusarium equiseti* D39	Fungal	unidentified plant, Yellow Sea, Qingdao, China	potent phytotoxicity,	47
Fusarisetin D (**74**)	*Fusarium equiseti* D39	Fungal	unidentified plant, Yellow Sea, Qingdao, China	potent phytotoxicity,	47
Pseurotin A (**75**)	*Aspergillus fumigatus* CUGBMF17018	Fungal	marine sediment, Bohai Sea, China	no antibacterial	97
	Aspergillus fumigatus MR2012	Fungal	Red Sea sediment, Hurghada, Egypt		89
	Bacillus sp. FS8D	Bacteria	marine animal Lepas anatifera	cytotox. vs. 4 glioma cells, IC_{50}=0.51–29.3μM	71
	Aspergillus fumigatus (030402d)	Fungal	deep-sea sediment, Vanuatu		67
	Aspergillus Fumigati	Fungal	sediment, Northeast Coast, Brazil		69
	Aspergillus fumigatus OUPS-T106B-5	Fungal	marine fish *Mugil cephalus*, Katsuura Bay, Japan		85

	Aspergillus sydowii PFW1-13	Fungal	driftwood sample, Baishamen, Hainan, China	significant antibacterial activities vs. *E. coli, B. subtilis, M. lysodeikticus*, MIC=14.49, 14.49, 7.24 μM	68
	Aspergillus fumigatus YK-7	Fungal	marine mud, Bo Hai, China	mod. cytotox. vs. U397, IC_{50}= 55. 9 μM	83
	Aspergillus fumigatus WFZ-25	Fungal	holothurian *Stichopus japonicus*, Lingshan Island, Qingdao, China		84
	Aspergillus fumigates	Fungal	giant jellyfish *Neopilema nomurai*, Korea		88
				monoamine oxidase inhibitory, apomorphine-antagonistic, chitin synthase inhibitory, induction of cell differentiation, nematicidal, immunosuppressive, antiparasitic and cytotox., antibacterial, antioxidant, osteoporosis inhibitor	72-82
11-*O*-methylpseurotin A (**76**)	*Aspergillus fumigatus* (030402d)	Fungal	deep-sea marine sediment, Vanuatu	selectively inhibited a Hof1 deletion yeast strain	67
	Aspergillus fumigatus MR2012		Red Sea sediment, Hurghada, Egypt		89
14-norpseurotin A (**77**)	*Aspergillus sydowii* PFW1-13	Fungal	driftwood sample (PFW1), Baishamen, Hainan, China,	significant antibacterial activities vs. *E. coli, B. subtilis*, and *M. ysoleikticus*, MIC= 3.74, 14.97, 7.49 μM	68
	Aspergillus fumigatus YK-7		marine mud, Bo Hai, China	mod. cytotox. U397, IC_{50}= 22. 8μM	83
	Phoma sp. NTOU4195		marine red alga *Pterocladiella capillacea*, Taiwan, China		70
	Aspergillus fumigates		giant jellyfish *Neopilema nomurai*, Korea		88
				Antiparasitic, cytotox. vs. MCF-7	78
Pseurotin A1 (**78**)	*Aspergillus fumigatus* WFZ-25	Fungal	holothurian *Stichopus japonicus*, Lingshan Island, Qingdao, China		84
	Aspergillus fumigatus OUPS-T106B-5		marine fish *Mugil cephalus*, Katsuura Bay, Japan		85
	Aspergillus fumigates		giant jellyfish *Neopilema nomurai*, Korea		88
	Aspergillus fumigatus YK-7		marine mud, Bo Hai, China	mod. cytotox. vs. U397, IC_{50}= 18. 5 μM	83

	Phoma sp. NTOU4195		marine red alga *Pterocladiella capillacea*, Taiwan, China	mod. anti-inflammatory	70
Pseurotin A2 (**79**)	*Aspergillus fumigatus* WFZ-25	Fungal	holothurian *Stichopus japonicus*, Lingshan Island, Qingdao, China	slightly cytotox.	84
	Aspergillus fumigatus MR2012		Red Sea sediment, Hurghada, Egypt	promising antiseizure	89
	Aspergillus fumigatus OUPS-T106B-5		marine fish *Mugil cephalus*, Katsuura Bay, Japan		85
	Aspergillus fumigates		giant jellyfish *Neopilema nomurai*, Korea		88
	Phoma sp. NTOU4195		marine red alga *Pterocladiella capillacea*, Taiwan, China	mod. anti-inflammatory	70
Pseurotin A3 (**80**)	*Phoma* sp. NTOU4195	Fungal	marine red alga *Pterocladiella capillacea*, Taiwan, China	mod. anti-inflammatory	70
Pseurotin G (**81**)	*Phoma* sp. NTOU4195	Fungal	marine red alga *Pterocladiella capillacea*, Taiwan, China	antiangiogenic, IC_{50}=16.7 μM, mod. anti-inflammatory	70
Pseurotin D (**82**)	*Aspergillus Fumigati*	Fungal	sediment, Northeast Coast of Brazil		69
				antagonistic, antiparasitic, and cytotox.	73,83
Pseurotin F2 (**83**)	*Phoma* sp. NTOU4195	Fungal	marine red alga *Pterocladiella capillacea*, Taiwan, China		70
				Antagonistic, chitin synthase inhibitory	74,86
Azaspirofuran A (**84**)	*Aspergillus fumigatus* MR2012	Fungal	Red Sea sediment, Hurghada, Egypt	promising antiseizure	89
	Aspergillus sydowii D2-6		marine sediments, Jiaozhou Bay, China	cytotox. vs. A549, IC_{50}=10 μM	87

Azaspirofuran B (**85**)	*Aspergillus fumigates*	Fungal	giant jellyfish *Neopilema nomurai*, Korea	NF	88
	Aspergillus fumigatus MR2012		Red Sea sediment, Hurghada, Egypt		89
	Aspergillus sydowii D2-6		marine sediments, Jiaozhou Bay, China		87
Pseurotin F1 (**86**)	*Aspergillus fumigates*	Fungal	giant jellyfish *Neopilema nomurai*, Korea	NF	88
	Aspergillus fumigatus MR2012		Red Sea sediment, Hurghada, Egypt		89
				antagonistic	69
Pseurotin G' (**87**)	*Aspergillus fumigatus* MR2012, co-culture	Fungal	Red Sea sediment, Hurghada, Egypt	ND	90
Cladosporicin A (**88**)	*Cladosporium sphaerospermum* SW67	Fungal	*Hydractinia echinata* (Cnidaria), a colony-forming hydrozoan, unknown place	weak cytotox. vs. 4 breast HCLs (70-90 μM)	91
Cephalimysin A (**89**)	*Aspergillus fumigatus* OUPS-T106B-5	Fungal	marine fish *Mugil cephalus*, Katsuura Bay, Japan,	significant cytotox. vs. P388 and HL-60 (IC_{50} =15.0, 9.5 nM).	92
Cephalimysin B (**90**)	*Aspergillus fumigatus* OUPS-T106B-5	Fungal	marine fish *Mugil cephalus*, Katsuura Bay, Japan,	NF	93
Cephalimysin C (**91**)	*Aspergillus fumigatus* OUPS-T106B-5	Fungal	marine fish *Mugil cephalus*, Katsuura Bay, Japan,	mod. cytotox. vs. P388 and HL-60 cell lines (IC_{50} 48–61μM)	93
Cephalimysin D (**92**)	*Aspergillus fumigatus* OUPS-T106B-5	Fungal	marine fish *Mugil cephalus*, Katsuura Bay, Japan,	mod. cytotox. vs. P388 and HL-60 cell lines (IC_{50} 48–61μM)	93
Cephalimysin E (**93**)	*Aspergillus fumigatus* OUPS-T106B-5	Fungal	marine fish *Mugil cephalus*, Katsuura Bay, Japan,	mod. cytotox. vs. 4 HTCLs (IC_{50} 34-59μM)	94-95
Cephalimysin F (**94**)	*Aspergillus fumigatus* OUPS-T106B-5	Fungal	marine fish *Mugil cephalus*, Katsuura Bay, Japan,	mod. cytotox. vs. 4 HTCLs (IC_{50} 55-63μM)	94-95
Cephalimysin G (**95**)	*Aspergillus fumigatus* OUPS-T106B-5	Fungal	marine fish *Mugil cephalus*, Katsuura Bay, Japan,	mod. cytotox. vs. 3 HTCLs (IC_{50} 15-59μM), KB, IC_{50}=11.1 μM, equal to 5-fluorouracil	94-95
Cephalimysin H (**96**)	*Aspergillus fumigatus* OUPS-T106B-5	Fungal	marine fish *Mugil cephalus*, Katsuura Bay, Japan,	mod. cytotox. vs. 3 HTCLs (IC_{50} 35-56μM), L1210, IC_{50}=12.8 μM	94-95

Cephalimysin I (**97**)	*Aspergillus fumigatus* OUPS-T106B-5	Fungal	marine fish *Mugil cephalus*, Katsuura Bay, Japan,	mod. cytotox. vs. 3 HTCLs (IC_{50} 31-69μM), L1210, IC_{50}=14.3 μM	94-95
Cephalimysin J (**98**)	*Aspergillus fumigatus* OUPS-T106B-5	Fungal	marine fish *Mugil cephalus*, Katsuura Bay, Japan,	mod. cytotox. vs. 4 HTCLs (IC50 51-58μM), KB–IC_{50}=7 μM, equal to 5-fluorouracil	94-95
Cephalimysin K (**99**)	*Aspergillus fumigatus* OUPS-T106B-5	Fungal	marine fish *Mugil cephalus*, Katsuura Bay, Japan,	mod. cytotox. vs. 4 HTCLs (IC_{50} 22-57μM)	94-95
Cephalimysin L (**100**)	*Aspergillus fumigatus* OUPS-T106B-5	Fungal	marine fish *Mugil cephalus*, Katsuura Bay, Japan,	mod. cytotox. vs. 4 HTCLs (IC_{50} 52-61μM)	94-95
Cephalimysin M (**101**)	*Aspergillus fumigatus* CUGBMF17018	Fungal	marine sediment, Bohai Sea, China	NF	97
Cephalimysin N (**102**)	*Aspergillus fumigatus* CUGBMF17018	Fungal	marine sediment, Bohai Sea, China	NF	97
FD-838 (**103**)	*Aspergillus Fumigati*	Fungal	sediment, Northeast Coast, Brazil		77
	Aspergillus fumigatus YK-7		marine mud, Bo Hai, China	mod. cytotox. vs. U397, IC_{50}= 25. 4μM	84
	Aspergillus fumigatus OUPS-T106B-5		marine fish *Mugil cephalus*, Katsuura Bay, Japan,	mod. cytotox. vs. P388 and HL-60 (IC_{50} 48–61 μM)	94-95
	Aspergillus fumigatus CUGBMF17018		marine sediment, Bohai Sea, China		97
				differentiation of leukemic cell, antibacterial and antifungal	96
Spirostaphylotrichin X (**104**)	*Cochliobolus lunatus* SCSIO41401	Fungal	marine alga, Yongxing Island, South China Sea,	obvious Anti-influenza multi-virus strains, IC_{50}= 1.2- 5.5μM, Targeting RNA Polymerase PB2	98
Spirostaphylotrichin A (**105**)	*Cochliobolus lunatus* SCSIO41401	Fungal	marine alga, Yongxing Island, South China Sea,	weak anti-influenzaa	98
Spirostaphylotrichin R (**106**)	*Cochliobolus lunatus* SCSIO41401	Fungal	marine alga, Yongxing Island, South China Sea,	NF	98
Triticone E (**107**)	*Cochliobolus lunatus* SCSIO41401	Fungal	marine alga, Yongxing Island, South China Sea,	NF	98
Triticone D (**108**)	*Westerdykella dispersa*	Fungal	Marine Sediment, South China Sea	NF	99
Ikarugamycin (**109**)	*Streptomyces zhaozhouensis* CA-185989	Actinomycete	marine sediment, Utonde, Equatorial Guinea	antifungal (MIC=4-8 μg/mL) and antibacterial vs. *MRSA* (MIC=2-4 μg/mL)	106

	Streptomyces *zhaozhouensis* subsp. *mycale*. subsp. nov MCCB267.		marine sponge, *Mycale* sp., *Rameswaram*, Southeast Coast, India	promising cytotox. vs. NCI-H460 (IC_{50}=1.43 μg/mL) via apoptosis	108
	Streptomyces xiamenensis 318		mangrove sediment, Fujian, China	cytotox. vs. pancreatic carcinoma, IC_{50} =1.30μM.	109
	Streptomyces sp. SCSIO 40060		marine sediment, South China Sea	mod. cytotox. vs. 3 HCLs, IC_{50}=1.96–6.87 μM.	110
				potent antiprotozoal, antibacterial (Gram-positive Bacteria, including *MRSA*), antifungal, antitumor activity, inhibition the uptake of oxidized low-density lipoprotein in macrophages, and inhibition HIV-1 Nef-induced CD4 cell surface downregulation, inhibition clathrin-dependent endocytosis	103-113
Butremycin (**110**)	Micromonospora sp. K310	Actinomycete	mangrove river sediment, Ghanaian	weak antibacterial	114
28-*N*-Methylikarugamycin (**111**)	*Streptomyces zhaozhouensis* CA-185989	Actinomycete	marine sediment, Utonde, Equatorial Guinea	antifungal (MIC=4-8μg/mL) and antibacterial vs. *MRSA* (MIC=1-2μg/mL)	106
	Streptomyces *zhaozhouensis* subsp. *mycale*. subsp. nov MCCB267.		marine sponge, *Mycale* sp., Rameswaram, Southeast Coast, India	promising cytotox. vs. NCI-H460 (IC_{50}= 1.78 μg/mL) via apoptosis	108
Isoikarugamycin (**112**)	*Streptomyces zhaozhouensis* CA-185989	Actinomycete	marine sediment, Utonde, Equatorial Guinea	antifungal (MIC=2-8μg/mL) and antibacterial vs. *MRSA* (MIC=2-4μg/mL)	106
30-Oxo-28-*N*-Methylikarugamycin (**113**)	Streptomyces *zhaozhouensis* subsp. *mycale*. subsp. nov MCCB267.	Actinomycete	marine sponge, *Mycale* sp., Rameswaram, Southeast Coast, India	promising cytotox. vs. NCI-H460 (IC_{50}= 7.17 μg/mL) via apoptosis	108
Clifednamide A (**114**)	Streptomyces *zhaozhouensis* subsp. *mycale*. subsp. nov MCCB267.	Actinomycete	marine sponge, *Mycale* sp., Rameswaram, Southeast Coast, India	promising cytotox. vs. NCI-H460 (IC_{50}= 16.29 μg/mL) via apoptosis	108
Capsimycin (**115**)	*Streptomyces* No. C 49-87	Actinomycete		antifungal	115
	Streptomyces xiamenensis 318		mangrove sediment, Fujian, China	cytotox. vs. pancreatic carcinoma, IC_{50}= 3.33μM.	109
	Streptomyces sp. SCSIO 40060		marine sediment, South China Sea	anti-*MRSA*, MIC = 16 mg/mL, cytotox. vs. several cancer cell lines, IC_{50}= 2.62– 6.87 μM	110
Epoxyikarugamycin/ Capsimycin B (**116**)	*Streptomyces xiamenensis* 318	Actinomycete	mangrove sediment, Fujian, China	cytotox. vs. pancreatic carcinoma, IC_{50} =3.37μM.	109

	Streptomyces sp. SCSIO 40060		marine sediment, South China Sea	mod. antimicrobial vs. 4 Bacterial MIC=8-16 μg/mL, mod. cytotox. vs. 3 HCLs, IC_{50}=1.96–6.87 μM.	110
				antibiotic activities vs. Gram-positive Bacteria and cytotox. vs. various HCLs.	105
Capsimycin C (**117**)	*Streptomyces xiamenensis* 318	Actinomycete	mangrove sediment, Fujian, China	weak cytotox.	109
	Streptomyces sp. SCSIO 40060		marine sediment, South China Sea		110
Capsimycin D (**118**)	*Streptomyces xiamenensis* 318	Actinomycete	mangrove sediment, Fujian, China	weak cytotox.	109
Capsimycin E (**119**)	*Streptomyces xiamenensis* 318	Actinomycete	mangrove sediment, Fujian, China	weak cytotox.	109
Capsimycin F (**120**)	*Streptomyces xiamenensis* 318	Actinomycete	mangrove sediment, Fujian, China	weak cytotox.	109
Capsimycin G (**121**)	*Streptomyces xiamenensis* 318	Actinomycete	mangrove sediment, Fujian, China	ND	109
Hydroxyikarugamycin A (**122**)	*Streptomyces* sp. SCSIO 40060	Actinomycete	marine sediment, South China Sea	NF	110
Hydroxyikarugamycin B (**123**)	*Streptomyces* sp. SCSIO 40060	Actinomycete	marine sediment, South China Sea	NF	110
Hydroxyikarugamycin C (**124**)	*Streptomyces* sp. SCSIO 40060	Actinomycete	marine sediment, South China Sea	NF	110
Chlokamycin (**125**)	*Streptomyces* sp. MA2-12	Actinomycete	Marine, unknown	mod. cytotox. vs. Jurkat cells, HCT116, IC_{50} =24.7, 33.5μM.	116
16-Hydroxymaltophilin (**126**)	*Actinoalloteichus cyanogriseus* WH1-2216-6	Actinomycete	marine sediment, China	mod. cytotox. vs. 6 HTCLs, IC_{50} between 4.5 to 9.7μM. No AF active. vs. *Aspergillus fumigatus*	117
Maltophilin (**127**)	*Actinoalloteichus cyanogriseus* WH1-2216-6	Actinomycete	marine sediment, China	mod. cytotox. vs. 6 HCLs IC_{50}=1.9 ~ 5.4μM, potent AF active. vs. *Aspergillus fumigatus* MIC=6.12μM (3.125 μg/mL)	117
Xanthobaccin C (**128**)	*Actinoalloteichus cyanogriseus* WH1-2216-6	Actinomycete	marine sediment, China	mod. cytotox. vs. 5 HCLs IC_{50}=3.4 ~ 7.0 μM, weak AF active. vs. *Aspergillus fumigatus* MIC=25 μg/mL)	117
Xanthobaccin C	Streptomyces CMB-CS038	Actinomycete	cone snails, unknown		100
FI-2 (**129**)	*Actinoalloteichus cyanogriseus* WH1-2216-6	Actinomycete	marine sediment, China	NF	117
Dihydromaltophilin/HSAF (**130**)	*Actinoalloteichus cyanogriseus* WH1-2216-6	Actinomycete	marine sediment, China	mod. cytotox. vs. 7 HCLs IC_{50}=0.1 ~ 4.9μM, potent AF activ. vs. *Aspergillus fumigatus* MIC=3.04μM (1.56 μg/mL)	117-119

	Streptomyces CMB-CS038		cone snails, unknown	antifungal vs. *Candida albicans* (IC_{50} 3 μM) and cytotox. vs. human colon (SW-620, IC_{50} 3.0 μM) and lung (NCI-H460, IC_{50} 5 μM) carcinoma cells	100
4-deoxy-dihydromaltophilin (**131**)	*Actinoalloteichus cyanogriseus* WH1-2216-6	Actinomycete	marine sediment, China	mod. cytotox. vs. 7 HCLs IC_{50}=0.4 ~ 5.7μM, weak AF active. vs. *Aspergillus fumigatus* MIC=25μg/mL)	117
F1-3 (**132**)	*Streptomyces* CMB-CS038	Actinomycete	cone snails, unknown	ND	100
$\triangle^{30}$-dihydromaltophilin (**133**)	*Streptomyces* CMB-CS038	Actinomycete	cone snails, unknown	ND	100
Pactamide A (**134**)	*Streptomyces* pactum SCSIO 02999	Actinomycete	marine sediment, South China Sea	Potent cytotox. vs. 4 HTCL, IC_{50} between 0.24 - 0.51μM	120
Pactamide B (**135**)	*Streptomyces* pactum SCSIO 02999	Actinomycete	marine sediment, South China Sea	Low cytotox. vs. 4 HTCL, IC_{50} between 21.9 - 26.1μM.	120
Pactamide C (**136**)	*Streptomyces* pactum SCSIO 02999	Actinomycete	marine sediment, South China Sea	Mod. cytotox. vs. 4 HTCL, IC_{50} between 0.71 - 2.42μM	120
Pactamide D (**137**)	*Streptomyces* pactum SCSIO 02999	Actinomycete	marine sediment, South China Sea	Low cytotox. vs. 4 HTCL, IC_{50} between 14.5 - 19.3μM.	120
Pactamide E (**138**)	*Streptomyces* pactum SCSIO 02999	Actinomycete	marine sediment, South China Sea	Mod. cytotox. vs. 4 HTCL, IC_{50} between 5.1 - 8.7μM	120
Pactamide F (**139**)	*Streptomyces* pactum SCSIO 02999	Actinomycete	marine sediment, South China Sea	Mod. cytotox. vs. 4 HTCL, IC_{50} between 2.6 - 2.9μM	120
Alteramide A (**140**)	*Alteromonas* sp.	Bacteria	Marine Sponge *Halichondria okadai*, Nagai, Kanagawa, Japan	cytotox.	121
Alteramide B (**141**)	*Alteromonas* sp.	Bacteria	Marine Sponge *Halichondria okadai*, Nagai, Kanagawa, Japan	NF	121
6-*epi*-Alteramide A (**142**)	*Pseudo Alteromonas* OT59	Bacteria	gorgonian octocoral (*Eunicea* sp.), Caribbean Panama, Pacific Ocean	dark-light-dependent antifungal	122
6-*epi*-Alteramide B (**143**)	*Pseudo Alteromonas* OT59	Bacteria	gorgonian octocoral (*Eunicea* sp.), Caribbean Panama, Pacific Ocean	dark-light-dependent antifungal	122
Aburatubolactam A (**144**)	Streptomyces sp. SCRC A-20	Actinomycete	mollusk, Aburatubo bay, Japan	antioxidant, potent inhibited superoxide anion generation	124
				cytotox., antimicrobial, and inhibition of superoxide generation	124-126

Aburatubolactam B (**145**)	Streptomyces sp. SCRC A-20	Actinomycete	mollusk, Aburatubo bay, Japan	antioxidant	124
Aburatubolactam C (**146**)	Streptomyces sp. SCRC A-20	Actinomycete	mollusk, Aburatubo bay, Japan	cytotox. vs. 8 tumor cells (IC_{50}=0.3-5.8μg/mL) via inducing apoptosis	125
Pyrrospirone C (**147**)	*Penicillium* sp. ZZ380	Fungal	wild sea crab *Pachygrapsus crassipes*, Unknown	anti-Bacterial activities vs. *MRSA* and *E. coli.* (MIC 4.0−5.0 μg/mL), mod. cytotox. vs. 4 glioma cells (IC_{50}=10.03–22.12 μM)	127
Pyrrospirone D (**148**)	*Penicillium* sp. ZZ380	Fungal	wild sea crab *Pachygrapsus crassipes*, Unknown	anti-Bacterial activities vs. *MRSA* and *E. coli.* (MIC 3−12 μg/mL), mod. cytotox. vs. 4 glioma cells (IC_{50}=9.95 –23.39μM)	127
Pyrrospirone E (**149**)	*Penicillium* sp. ZZ380	Fungal	wild sea crab *Pachygrapsus crassipes*, Unknown	anti-Bacterial activities vs. *MRSA* and *E. coli.* (MIC 10−11 μg/mL), mod. cytotox. vs. 4 glioma cells (IC_{50}=15.76 –26.64μM)	127
	Penicillium sp. CPCC 400817		mangrove plant, Dongzhai harbour, Hainan, China	potent antibacterial vs. methicillin-susceptible and methicillin-resistant *Staphylococcus aureus*, MIC =12.9, 12.9 μg/mL	130
Pyrrospirone F (**150**)	*Penicillium* sp. ZZ380	Fungal	wild sea crab *Pachygrapsus crassipes*, Unknown	anti-Bacterial activities vs. *MRSA* and *E. coli.* (MIC 2.0−3.0 μg/mL), mod. cytotox. vs. 4 glioma cells (IC_{50}=7.44–19.18 μM)	127
	Penicillium sp. CPCC 400817		mangrove plant, Dongzhai harbour, Hainan, China	potent antibacterial vs. methicillin-susceptible and methicillin-resistant *Staphylococcus aureus*, MIC =25.8, 25.8 μg/mL	130
Pyrrospirone G (**151**)	*Penicillium* sp. ZZ380	Fungal	wild sea crab *Pachygrapsus crassipes*, Unknown	potent cytotox. vs. 4 glioma cells (IC_{50}=1.06–8.52 μM)	127
Pyrrospirone H (**152**)	*Penicillium* sp. ZZ380	Fungal	wild sea crab *Pachygrapsus crassipes*, Unknown	anti-Bacterial activities vs. *MRSA* and *E. coli.* (MIC 4−19 μg/mL), mod. cytotox. vs. 4 glioma cells (IC_{50}=12.89–23.92 μM)	127
Pyrrospirone I (**153**)	*Penicillium* sp. ZZ380	Fungal	wild sea crab *Pachygrapsus crassipes*, Unknown	anti-Bacterial activities vs. *MRSA* and *E. coli.* (MIC 2.0−4 μg/mL), mod. cytotox. vs. 4 glioma cells (IC_{50}=7.44–19.18 μM)	127
Pyrrospirone J (**154**)	*Penicillium* sp. ZZ380	Fungal	wild sea crab *Pachygrapsus crassipes*, Unknown	potent cytotox. vs. 2 human glioma cells, IC_{50}=10.52–17.92 μM	129
Penicipyrrodiether A (**155**)	*Penicillium* sp. ZZ380	Fungal	wild sea crab *Pachygrapsus crassipes*, Unknown	anti-Bacterial activities vs. *MRSA* (MIC=5.0 μg/mL), and E. coli (MIC=34 μg/mL), mod. anti-glioma, IC_{50} = 11.32 –29.10μM,	128
Penicipyrroether A (**156**)	*Penicillium* sp. ZZ380	Fungal	wild sea crab *Pachygrapsus crassipes*, Unknown	potent, selective cytotox. vs. 2 human glioma cells, IC_{50}=1.64–5.50 μM, slightly stronger than p.c. Doxorubicin and antibacterial, MIC=1.7 μg/mL vs. *MRSA* and 3.0 μg/mL vs. *E. coli*	129
GKK1032A2 (**157**)	*Penicillium* sp. CPCC 400817	Fungal	mangrove plant, Dongzhai harbour, Hainan, China	potent antibacterial vs. methicillin-susceptible and methicillin-resistant *Staphylococcus aureus*, MIC =3.2, 3.2 μg/mL	130
GKK1032B (**158**)	*Penicillium* sp. CPCC 400817	Fungal	mangrove plant, Dongzhai harbour, Hainan, China	potent antibacterial vs. methicillin-susceptible and methicillin-resistant *Staphylococcus aureus*, MIC =3.2, 25.8 μg/mL	130

GKK1032C (**159**)	*Penicillium* sp. CPCC 400817	Fungal	mangrove plant, Dongzhai harbour, Hainan, China	potent antibacterial vs. methicillin-susceptible and methicillin-resistant *Staphylococcus aureus*, MIC =3.2, 1.6 μg/mL	130
Trichobamide A (**160**)	*Trichobotrys effuse* 4729	Fungal	ascidian, South China Sea	significant cytotox., anti-glioma	131
Ascomylactam A (**161**)	*Didymella* sp. CYSK-4	Fungal	semi-mangrove *Pluchea indica*, Guangxi, China	mod. cytotox. vs. 6 HTCLs, IC_{50} values in the range of 4.4-6.8 μM.	133
Ascomylactam B (**162**)	*Didymella* sp. CYSK-4	Fungal	semi-mangrove *Pluchea indica*, Guangxi, China	mod. cytotox. vs. 6 HTCLs, IC_{50} values in the range of 4.5-20 μM.	133
Ascomylactam C (**163**)	*Didymella* sp. CYSK-4	Fungal	semi-mangrove *Pluchea indica*, Guangxi, China	mod. cytotox. vs. 6 HTCLs, IC_{50} values in the range of 4.2−7.8 μM.	133
Phomapyrrolidone A (**164**)	*Didymella* sp. CYSK-4	Fungal	semi-mangrove *Pluchea indica*, Guangxi, China	mod. cytotox. vs. 6 HTCLs, IC_{50} values in the range of 12-29 μM.	133
				very weak antitubercular (MABA, LORA Test MIC=20.1, 41.1 μM) at subcytotox. concentrations.	132
Phomapyrrolidone C (**165**)	*Didymella* sp. CYSK-4	Fungal	semi-mangrove *Pluchea indica*, Guangxi, China	mod. cytotox. vs. 4 HTCLs, IC_{50} values in the range of 25−30 μM.	133
				weak antitubercular (MABA, LORA Test MIC=5.2, 13.4 μM) at subcytotox. concentrations.	132
Jamaicamide A (**166**)	*Moorea producens* JHB	Cyanobacteria	Hector Bay, Jamaica	Cytotox. vs. 2 HTCLs LC_{50}=15 μM, sodium channel-blocking 5μM	135
	Moorea producens JHB		Hector Bay, Jamaica	concentration-dependent antagonism for the increase in neuronal [Ca2+]i/[Na+]i induced by veratridine, IC_{50}=1.82, 1.1μM	136
Jamaicamide B (**167**)	*Moorea producens* JHB	Cyanobacteria	Hector Bay, Jamaica	neurotoxic 5 ppm. Cytotox. vs. 2 HTCLs LC_{50}=15μM,sodium channel-blocking 5μM	135
	Moorea producens JHB		Hector Bay, Jamaica	concentration-dependent antagonism for the increase in neuronal $[Ca^{2+}]i/[Na^{+}]i$ induced by veratridine, IC_{50}=6.88, 3.6μM	136
Jamaicamide C (**168**)	*Moorea producens* JHB	Cyanobacteria	Hector Bay, Jamaica	neurotoxic 10 ppm. Cytotox. vs. 2 HTCLs LC_{50}=15 μM sodium channel-blocking 5μM	135
Jamaicamide D (**169**)	*Moorea producens* JHB	Cyanobacteria	Hector Bay, Jamaica	ND	136
Jamaicamide E (**170**)	*Moorea producens* JHB	Cyanobacteria	Hector Bay, Jamaica	ND	136
Jamaicamide F (**171**)	*Moorea producens* JHB	Cyanobacteria	Hector Bay, Jamaica	concentration-dependent antagonism for the increase in neuronal $[Ca^{2+}]i/[Na^{+}]i$ induced by veratridine, IC_{50}= 4.3, 2.3μM	136
Microcolin A (**172**)	*Lyngbya majuscula*	Cyanobacteria	shallow-water, Venezuelan	immunosuppressant EC_{50}=1.5nM	137
	Lyngbya cf. polychroa		shallow-water, Hollywood, Florida, USA	cytotox. vs. HT-29, IMR-32, IC_{50}= 0.28, 0.31nM	138

	Moorea producens		shallow-water, Playa Kalki, Curacao, Holland	significant cytotox. vs. H-460, IC_{50}=6, 5.0μM	139,141
Microcolin B (**173**)	*Lyngbya majuscula*	Cyanobacteria	shallow-water, Venezuelan	immunosuppressant EC50=42.7nM	137
				inhibit LFA-A/ICAM-1 meditated cell adhesion IC_{50}= 0.15 μM	140
	Lyngbya cf. polychroa		shallow-water, Hollywood, Florida, USA	inhibit the growth of HT-29 and IMR-32, IC_{50}= 2.3, 7.7nM	138
	Moorea producens		shallow-water, Playa Kalki, Curacao, Holland	significant cytotox. vs. H-460, IC_{50}=6, 5.0μM	139
Desacetylmicrocolin B /Microcolin C (**174**)	Lyngbya cf. polychroa	Cyanobacteria	shallow-water, Hollywood, Florida, USA	inhibit the growth of HT-29 and IMR-32, IC_{50}= 14nM	138
	Moorea producens		shallow-water, Playa Kalki, Curacao, Holland	significant cytotox. vs. H-460, IC_{50}=6, 5.0μM	139
Microcolin D (**175**)	*Moorea producens*	Cyanobacteria	shallow-water, Playa Kalki, Curacao, Holland	significant cytotox. vs. H-460, IC_{50}=6, 5.0μM	139
				inhibit LFA-A/ICAM-1 meditated cell adhesion IC_{50}= 0.9μM	140
Microcolin E (**176**)	*Moorea producens*	Cyanobacteria	shallow-water, Playa Kalki, Curacao, Holland	significant cytotox. vs. H-460, IC_{50}=6, 5.0μM	139
Microcolin F (**177**)	*Moorea producens*	Cyanobacteria	shallow-water, Playa Kalki, Curacao, Holland	significant cytotox. vs. H-460, IC_{50}=6, 5.0μM	139
Microcolin G (**178**)	*Moorea producens*	Cyanobacteria	shallow-water, Playa Kalki, Curacao, Holland	significant cytotox. vs. H-460, IC_{50}=6, 5.0μM	139
Microcolin H (**179**)	*Moorea producens*	Cyanobacteria	shallow-water, Playa Kalki, Curacao, Holland	significant cytotox. vs. H-460, IC_{50}=6, 5.0μM	139
Microcolin I (**180**)	*Moorea producens*	Cyanobacteria	shallow-water, Playa Kalki, Curacao, Holland	significant cytotox. vs. H-460, IC_{50}=6, 5.0μM	139
Microcolin J (**181**)	*Moorea producens*	Cyanobacteria	shallow-water, Playa Kalki, Curacao, Holland	significant cytotox. vs. H-460, IC_{50}=6, 5.0μM	139
Microcolin K (**182**)	*Moorea producens*	Cyanobacteria	shallow-water, Playa Kalki, Curacao, Holland	significant cytotox. vs. H-460, IC_{50}=6, 5.0μM	139
Microcolin L (**183**)	*Moorea producens*	Cyanobacteria	shallow-water, Playa Kalki, Curacao, Holland	significant cytotox. vs. H-460, IC_{50}=6, 5.0μM	139
Majusculamide D (**184**)	*Moorea* sp.	Cyanobacteria	shallow-water, agoon of Enewetak AtollPapua New Guinea	absolute configuration, selective and potent cytotox. (PANC-1 and U251N, IC_{50}= 0.32, 36.8nM)	143

	Lyngbya majuscula		shallow-water, agoon of Enewetak Atoll, Papua New Guinea	mod. cytotox. vs. CCRF-CEM cell culture system at 0.2 pg/mL.	142
Deoxymajusculamide D (**185**)	*Lyngbya majuscula*	Cyanobacteria	shallow-water, Marshall Islands, Western Pacific	mod. cytotox. in the CCRF-CEM cell culture system at 0.2 pg/mL.	142
Ypaoamide (**186**)	*Lyngbya majuscula*	Cyanobacteria	Guam, Japan	antifeedant, broadly acting feeding deterrent	144
Ypaoamides B (**187**)	*Okeania* sp.	Cyanobacteria	shallow-water, Okinawa, Japan	antidiabetic, stimulated glucose uptake in cultured rat L6 myotubes via AMPK pathway	145
Ypaoamides C (**188**)	*Okeania* sp.	Cyanobacteria	shallow-water, Okinawa, Japan	antidiabetic, stimulated glucose uptake in cultured rat L6 myotubes.	145
Palmyrrolinone (**189**)	Cyanobacterial assemblage	Cyanobacteria	Palmyra Atoll,	molluscicidal	146
Malyngamide A (**190**)	*Moorea producens*	Cyanobacteria	shallow-water, Red Sea, Egypt	weak cytotox.	153
	Lyngbya majuscula		shallow-water, Kahala Beach, Oahu, Hawaii, USA		147-148
Malyngamide B (**191**)	*Moorea producens*	Cyanobacteria	shallow-water, Red Sea, Egypt	mod. cytotox.	153
	Lyngbya majuscula		shallow-water, Kahala Beach, Oahu, Hawaii, USA		147-148
Pukeleimide A (**192**)	*Lyngbya majuscula*	Cyanobacteria	shallow-water, Kahala Beach, Oahu, USA	NF	150
Pukeleimide B (**193**)	*Lyngbya majuscula*	Cyanobacteria	shallow-water, Kahala Beach, Oahu, USA	NF	150
Pukeleimide C (**194**)	*Lyngbya majuscula*	Cyanobacteria	shallow-water, Kahala Beach, Oahu, USA	NF	149
Pukeleimide D (**195**)	*Lyngbya majuscula*	Cyanobacteria	shallow-water, Kahala Beach, Oahu, USA	NF	150
Pukeleimide E (**196**)	*Lyngbya majuscula*	Cyanobacteria	shallow-water, Kahala Beach, Oahu, USA	NF	150
Pukeleimide F (**197**)	*Lyngbya majuscula*	Cyanobacteria	shallow-water, Kahala Beach, Oahu, USA	NF	150
Pukeleimide G (**198**)	*Lyngbya majuscula*	Cyanobacteria	shallow-water, Kahala Beach, Oahu, USA	NF	150
Malyngamide Q (**199**)	*Lyngbya majuscula*	Cyanobacteria	shallow-water, madagascan	unstanble, NF	151

Malyngamide R (**200**)	*Lyngbya majuscula*	Cyanobacteria	shallow-water, madagascan	modestly brine-shrimp toxic (LD_{50}=18 ppm)	151
Isomalyngamide A (**201**)	*Lyngbya majuscula*	Cyanobacteria	Hawaiian waters, USA	lethal toxicity to crayfish, IC_{50}= 250 μg/kg	152
	Lyngbya majuscula		Taiwan, China	significantly suppressive effects on tumor migration (serum-induced MDA-MB-231 IC_{50}=60nM) rather than proliferation (IC_{50}=4.6μM), inhibition of b1-integrin hypersialylation.	154
	Lyngbya majuscula		Hawaiian waters, USA	cytotox. vs. L1210, IC_{50}=130 μM, and lethal toxicity vs. the shrimp *Palaemon paucidens* at an LD100 =4.25 mg/kg.	155
Isomalyngamide B (**202**)	*Lyngbya majuscula*	Cyanobacteria	Hawaiian waters, USA	lethal toxicity to crayfish at 500 μg/kg	155
	Lyngbya majuscula		Hawaiian waters, USA	cytotox. vs. L1210, IC_{50}=30 μM and lethal toxicity vs. the shrimp *Palaemon paucidens* at an LD100 = 1.7 mg/kg.	152
Malyngamide 4 (**203**)	*Moorea producens*	Cyanobacteria	shallow-water, Red Sea, Egypt	mod. cytotox.`	153
Isomalyngamide A-1 (**204**)	*Lyngbya* majuscula	Cyanobacteria	Taiwan, China	significantly suppressive effects on tumor migration (serum-induced MDA-MB-231 IC_{50}=337nM) rather than proliferation (IC_{50}=12.7μM), inhibition of b1-integrin hypersialylation.	154
New Malyngamide (**205**)	*Lyngbya majuscula*	Cyanobacteria	Hawaiian waters, USA	cytotox. vs. L1210, IC_{50}=2.9 μM and lethal toxicity vs. the shrimp *Palaemon paucidens* at an LD100 = 33.3 mg/kg.	155
Palau'Imide (**206**)	Lyngbya sp.	Cyanobacteria	shallow-water, Palau, Japan	cytotox. vs. KB, d LoVo cells, IC_{50}= 1.4, 0.36 mM	156
Belamide A (**207**)	*Symploca* sp.	Cyanobacteria	Florida	mod. cytotox. vs. HCT-116 (IC_{50}=0.74μM), microtubule depolymerizing effects in A-10 cells at 20 μM	157
Caldoramide (**208**)	*Caldora penicillata*	Cyanobacteria	Salmedina Reef, Panama	mod. cytotox. for cells containing both oncogenic KRAS and HIF over the corresponding knockout cells.	158
Gallinamide A/Symplostatin 4 (**209**)	*Schizothrix*	Cyanobacteria	tropical reef, Piedras Gallinas, Caribbean Coast of Panama	antimalarial	159-164
	Symploca sp.		shallow-water, Key Largo (Florida Keys), USA	cytotox., inhibits the human cysteine protease cathepsin L, inhibitor of FPs in infected RBCs	159-164
Cyclopiazonic Acid (**210**)	*Aspergillus flavus* C-F-3	Fungal	marine algae, Putian Pinghai, China	cytotox. vs. 4 HTCLs (IC_{50}=2.4–21.5μM)	169
	Aspergillus flavus OUCMDZ-2205		Prawn, *Penaeus vannamei,* Lianyungang sea, Jiangsu, China		170
	Penicillium vinaceum		sponge *Hyrtios erectus* Yanbu, Red Sea, Egypt	only antibacterial vs. E. coli and showed inhibition zone of 20 mm	184

	Penicillium commune DFFSCS026		deep-sea sediment, South China Sea	toxicity to brine shrimp, IC_{50}<1.0ug/mL	181
	Penicillium cyclopium Westling			most notably Ca^{2+}-ATPase inhibition	166
				immunosuppressive, antiviral activities vs. Sendai virus, hepatitis B virus, otavirus and human respiratory syncytial virus	167-168
cAATrp (**211**)	*Aspergillus oryzae* HMP-F28	Fungal	marine sponge *Hymeniacidon perleve*, Bohai Sea, China	ND	174
β-CPA (**212**)	*Aspergillus oryzae* HMP-F28	Fungal	marine sponge *Hymeniacidon perleve*, Bohai Sea, China	ND	174
Iso-*α*-Cyclopiazonic acid (**213**)	*Aspergillus flavus* C-F-3	Fungal	marine algae, Putian Pinghai, China	cytotox. vs. A549 (IC_{50}=42.2μM)	169
	Aspergillus flavus OUCMDZ-2205		Prawn, *Penaeus vannamei*, Lianyungang sea, Jiangsu, China		170
Amycocyclopiazonic acid (**214**)	Amycolatopsis sp	Actinomycete	sponge, *Micronesia*, Western Pacific	NF	172
Amycolactam (**215**)	Amycolatopsis sp	Actinomycete	sponge, *Micronesia*, Western Pacific	significant cytotox. vs. SNU638, HCT116 (IC_{50}=0.8, 2.0 μM), mod. cytotox. vs. A546, K562, SK-HEP1 (IC_{50} = 13.7, 9.6, 8.3μM)	172
Pseuboydone E (**216**)	*Pseudallescheriaboydii* F19-1	Fungal	soft coral *Lobophytum crassum*, Hainan Sanya, China	ND	173
Cyclopiamide (**217**)	*Aspergillus flavus* MXH-X104	Fungal	sponge *Agelas aff. nemoechinata*, Xisha Islands, China	_	179
	Penicillium commune DFFSCS026		deep-sea sediment, South China Sea	weak toxicity to brine shrimp (IC_{50}=14.1–46.5 ug/mL), inactive vs. 2 HTCLs and H1N1 virus	181
Cyclopiamide B (**218**)	*Penicillium commune* DFFSCS026	Fungal	deep-sea sediment, South China Sea	weak toxicity to brine shrimp (IC_{50}=14.1–46.5 ug/mL), inactive vs. 2 HTCLs and H1N2 virus	181
Cyclopiamide C (**219**)	*Penicillium commune* DFFSCS026	Fungal	deep-sea sediment, South China Sea	weak toxicity to brine shrimp (IC_{50}=14.1–46.5 ug/mL), inactive vs. 2 HTCLs and H1N3 virus	181
Cyclopiamide D (**220**)	*Penicillium commune* DFFSCS026	Fungal	deep-sea sediment, South China Sea	weak toxicity to brine shrimp (IC_{50}=14.1–46.5 ug/mL), inactive vs. 2 HTCLs and H1N4 virus	181

Cyclopiamide E (**221**)	*Penicillium commune* DFFSCS026	Fungal	deep-sea sediment, South China Sea	weak toxicity to brine shrimp (IC_{50}=14.1–46.5 ug/mL), inactive vs. 2 HTCLs and H1N5 virus	181
Cyclopiamide F (**222**)	*Penicillium commune* DFFSCS026	Fungal	deep-sea sediment, South China Sea	weak toxicity to brine shrimp (IC_{50}=14.1–46.5 ug/mL), inactive vs. 2 HTCLs and H1N6 virus	181
Cyclopiamide G (**223**)	*Penicillium commune* DFFSCS026	Fungal	deep-sea sediment, South China Sea	weak toxicity to brine shrimp (IC_{50}=14.1–46.5 ug/mL), inactive vs. 2 HTCLs and H1N7 virus	181
Cyclopiamide H/Sperade B Speradine G (**224**)	*Penicillium commune* DFFSCS026	Fungal	deep-sea sediment, South China Sea	NF	181
	Aspergillus oryzae		marine sediment, Min River, China	NF	178
	Aspergillus oryzae		marine sediments, Langqi Island, Fujian, China	very weak cytotox. vs. HeLa IC_{50} = 0.2 mM	177
Cyclopiamide I/Aspergilline D (**225**)	*Penicillium commune* DFFSCS026	Fungal	deep-sea sediment, South China Sea	weak toxicity vs. brine shrimp (IC_{50}=14.1–46.5 ug/mL), inactive vs. 2 HTCLs and H1N8 virus	181
	Aspergillus versicolor			Anti-TMV vs. Nicotiana tabacum Leaf 38.9μM, protective effect on the host plant, mod. cytotox. 5 HTCLs, IC_{50}= 1.2 - 4.2 μM	182
Cyclopiamide J (**226**)	*Penicillium commune* DFFSCS026	Fungal	deep-sea sediment, South China Sea	weak toxicity to brine shrimp (IC_{50}=14.1–46.5 ug/mL), inactive vs. 2 HTCLs and H1N8 virus	181
Speradine A (**227**)	*Aspergillus tamarii* M143	Fungal	driftwood, Okinawa, Japan	inhibitory vs. Ca^{2+}-ATPase (IC_{50} 8 μM), inhibitory vs. histone deacetylase (IC_{50} 100 ug/mL), and antibacterial vs. *Mycrococcus luteus* (MIC 16.7 ug/mL).	175
3-OH-Speradine A (**228**)	*Aspergillus oryzae* HMP-F28	Fungal	marine sponge Hymeniacidon perleve, Bohai Sea, China	significant extracellular alkalinization coupled, H2O2 production in tobacco suspensions. lethality-toxicity of brine shrimp LC_{50}=39.7ug/mL, mod. cytotox. IC_{50}=29.6-43.3μM, promoting plant growth	174
Speradine C (**229**)	*Aspergillus oryzae*	Fungal	marine sediments, Langqi Island, Fujian, China	NF	177
	Aspergillus flavus MXH-X104		sponge *Agelas* aff. *nemoechinata*, Xisha Islands, China	NF	179
Speradine D (**230**)	*Aspergillus oryzae*	Fungal	marine sediments, Langqi Island, Fujian, China	NF	177
Speradine E (**231**)	*Aspergillus oryzae*	Fungal	marine sediments, Langqi Island, Fujian, China	very weak cytotox. vs. HeLa, IC_{50} = 0.2 mM	177
	Penicillium commune DFFSCS026		deep-sea sediment, South China Sea	NF	181

	Aspergillus oryzae		marine sediment, Min River, China	weak toxicity vs. brine shrimp, IC_{50}=46.5 ug/mL	178
Speradine F-Speradine B Penicamedine A (**232**)	*Pseudallescheriaboydii* F19-1	Fungal	soft coral *Lobophytum crassum*, Hainan Sanya, China	NF	173
	Aspergillus flavus MXH-X104		sponge *Agelas aff. nemoechinata*, Xisha Islands, China	NF	179
	Penicillium camemberti		unknown, southwestern Pacific Ocean	NF	171
2-Demethylsperadine F-Speradine B (**233**)	*Penicillium* dipodomyicola Y26-02	Fungal	mangrove *Clerodendrum inerme,* south China Sea	weak anti-HIV	180
Speradine H (**234)**	*Aspergillus oryzae*	Fungal	marine sediment, Min River, China	NF	178
	Penicillium commune DFFSCS026		deep-sea sediment, South China Sea	weak toxicity vs. brine shrimp (IC_{50}=28.9 ug/mL)	181
Speradine C'-Speradine C (**235**)	*Pseudallescheriaboydii* F19-1	Fungal	soft coral *Lobophytum crassum*, Hainan Sanya, China	significant cytotox. vs. Sf9 insect cells, IC_{50} = 0.9 μM	173
	Aspergillus flavus MXH-X104		sponge *Agelas aff. nemoechinata*, Xisha Islands, China	NF	179
Pyranonigrin A (**236**)	*Aspergillus niger Van Tieghem*	Fungal	sponge *Axinella damicornis*, Mediterranean	NF	185,188-191
	Aspergillus niger LL-LV3020		mangrove wood, Hong Kong, China	Revised	186
	Penicillium brocae MA-231		mangrove plants *Avicennia marina*, Hainan, China	potent antibacterial vs. 7 human-, aqua-, and plant-pathogens (MIC=0.5μg/mL)	187
Pyranonigrin B (**237**)	*Aspergillus niger Van Tieghem*	Fungal	sponge *Axinella damicornis*, Mediterranean	NF	185
Pyranonigrin C (**238**)	*Aspergillus niger Van Tieghem*	Fungal	sponge *Axinella damicornis*, Mediterranean	NF	185
Pyranonigrin D (**239**)	*Aspergillus niger Van Tieghem*	Fungal	sponge *Axinella damicornis*, Mediterranean	ND	185

Pyranonigrin F (**240**)	*Penicillium brocae* MA-231	Fungal	mangrove *Avicennia marina*, Hainan, China	potent antibacterial vs. 7 human-, aqua-, and plant-pathogens (MIC=0.5μg/mL)	187
Pyranonigrin S (**241**)	*Aspergillus niger* LL-LV3020	Fungal	mangrove wood, Hongkong, China	antioxidant	186,188,190,191
Nigrospine (**242**)	*Nigrospora oryzae* SCSGAF 0111	Fungal	coral gorgonian *Verrucellaum braculum*, South China Sea	ND	192
Vermelhotin (**243**)	Pleosporale CRI247-01	Fungal	sponge, Surin Island, Phangnga Province, Thailand	cytotox. vs. 11 HCLs (0.31–13.5μg/mL), mod. antiplasmodial (MIC 1-10 μM). calmodulin inhibitor, anti-inflammatory, anti-TB	194-197
Cladosporiumin J (**244**)	*Cladosporium sphaerospermum* EIODSF 008	Fungal	deep-sea sediment, East Indian Ocean	NF	32
Cladosporiumin K (**245**)	*Cladosporium sphaerospermum* EIODSF 008	Fungal	deep-sea sediment, East Indian Ocean	NF	32
Cladosporiumin I (**246**)	*Cladosporium sphaerospermum* EIODSF 008	Fungal	deep-sea sediment, East Indian Ocean	NF	32
Cladodionen (**247**)	*Cladosporium* sp. OUCMDZ-1635	Fungal	sponge, Xisha Islands, South China Sea	cytotox. vs. MCF-7, HeLa, HCT-116, and HL-60 s, (IC_{50} of 18.7, 19.1, 17.9, 9.1μM)	198
	Cladosporium sphaerospermum L3P3		deep-sea sediment, Mariana Trench	cytotox. vs. HL-60, K562 (IC_{50}=4.5, 6.6 μM)	201
Cladosporiumin A (**248**)	*Cladosporium* sp. SCSIO z0025	Fungal	deep-sea sediment, Okinawa Trough, Japan	NF	31
Cladosporiumin B (**249**)	*Cladosporium* sp. SCSIO z0025	Fungal	deep-sea sediment, Okinawa Trough, Japan	NF	31
Cladosporiumin C (**250**)	*Cladosporium* sp. SCSIO z0025	Fungal	deep-sea sediment, Okinawa Trough, Japan	NF	31
Cladosporiumin D (**251**)	*Cladosporium* sp. SCSIO z0025	Fungal	deep-sea sediment, Okinawa Trough, Japan	NF	31
Cladosporiumin I' (**252**)	*Cladosporium sphaerospermum* SW67	Fungal	*Hydractinia echinata* (Cnidaria), a colony-forming hydrozoan, unknown place	weak cytotox. vs. 4 breast HCLs (70-90 μM)	91
Cladosporiumin J' (**253**)	*Cladosporium sphaerospermum* SW67	Fungal	*Hydractinia echinata* (Cnidaria), a colony-	weak cytotox. vs. 4 breast HCLs (70-90 μM)	91

			forming hydrozoan, unknown place		
Cladosin A (**254**)	*Cladosporium sphaerospermum* 2005-01-E3	Fungal	deep-sea sediment, Pacific Ocean	NF	199
Cladosin B (**255**)	*Cladosporium sphaerospermum* 2005-01-E3	Fungal	deep-sea sediment, Pacific Ocean	NF	199
Cladosin C (**256**)	*Cladosporium sphaerospermum* 2005-01-E3	Fungal	deep-sea sediment, Pacific Ocean	mild anti-virus (influenza A H1N1)	199
Cladosin D (**257**)	*Cladosporium sphaerospermum* 2005-01-E3	Fungal	deep-sea sediment, Pacific Ocean	NF	199
Cladosin F (**258**)	*Cladosporium sphaerospermum* 2005-01-E3	Fungal	deep-sea sediment, Pacific Ocean	NF	199
Cladosin G (**259**)	*Cladosporium sphaerospermum* 2005-01-E3	Fungal	deep-sea sediment, Pacific Ocean	NF	199
Cladosin H (**260**)	*Cladosporium sphaerospermum* L3P3	Fungal	deep-sea sediment, Mariana Trench	NF	201
Cladosin I (**261**)	*Cladosporium sphaerospermum* L3P3	Fungal	deep-sea sediment, Mariana Trench	cytotox. vs. HL-60/K562 (IC_{50} =2.8 /4.1 μM)	201
Cladosin J (**262**)	*Cladosporium sphaerospermum* L3P3	Fungal	deep-sea sediment, Mariana Trench	cytotox. vs. HL-60/K562 (IC_{50}= 6.8 /7.8 μM)	201
Cladosin K (**263**)	*Cladosporium sphaerospermum* L3P3	Fungal	deep-sea sediment, Mariana Trench	cytotox. vs. HL-60/K562 (IC_{50}=5.9 /7.5 μM)	201
Tersone F (**264**)	*Phomopsis tersa* fs441	Fungal	deep-sea sediment, Indian Ocean	NF	202
Lajollamycin (**265**)	Streptomyces *nodosus* (NPS007994)	Actinomycete	marine sediment, Scripps, California, USA	antibacterial vs. both drug-sensitive and -resistant Gram-positive Bacteria, cytotox. inhibited the growth of B16-F10 (EC50=9.6 μM)	203
	Streptomyces sp. (SMC72)		seashore sediment, Jeju Island, KoreAspergillus	mod. antifungal vs. *Candida albicans* IC_{50}= 42 μM	204
Lajollamycin B (**266**)	*Streptomyces* sp. (SMC72)	Actinomycete	seashore sediment, Jeju Island, KoreAspergillus	mod. antifungal vs. *Candida albicans* IC_{50}= 40 μM,	204
Lajollamycin C (**267**)	*Streptomyces* sp. (SMC72)	Actinomycete	seashore sediment, Jeju Island, KoreAspergillus	mod. antifungal vs. *Candida albicans* IC_{50}=50 μM,	204
Lajollamycin D (**268**)	*Streptomyces* sp. (SMC72)	Actinomycete	seashore sediment, Jeju Island, KoreAspergillus	mod. antifungal vs. *Candida albicans* IC_{50}= 120 μM,	204

Streptopyrrolidine (**269**)	*Streptomyces* sp. KORDI-3973	Actinomycete	deep-sea sediment, Ayu Trough, western Pacific	anti-angiogenesis	205
Spinoxazine A (**270**)	*Streptomyces spinoverrucosus*	Actinomycete	sand, Bahamas	NF	206
Epolactaene (**271**)	*Penicillium* sp. BM1689-P	Fungal	sea sediment, Japan	neuritogenic arrests the cell cycle at the G0/G1 phase, induces the outgrowth of neurites in human neuroblastoma SH-SY5Y.	207-208
				binds to Hsp60, inhibits Hsp60 chaperone, cytotox. induces apoptosis in a human leukemia B, BALL-1	209-210
Pulchellalactam (**272**)	*Corollospora pulchella*	Fungal	driftwood, Peleliu, Japan	CD45 Protein Tyrosine Phosphatase Inhibitor	211
Hoshinolactam (**273**)	*Oscillatoria*	Cyanobacteria	Coast near Hoshino, Okinaw, Japan	potent antitrypanosomal (IC_{50}=3.9 nM), out cytotox. vs. MRC-5 (IC_{50} > 25μM).	212
3- (2- Amino-Phenyl)-5-Methoxy-1, 5-Dihydro-Pyrrol-2-One (**274**)	*Rapidithrix thailandica* strain TISTR 1741	Bacteria	biofilm specimen, Andaman Sea, Thailand	Antibacterial, selective inhibition vs. VRE (MIC = 5.97 μM)	213
Andrimid (**275**)	*Pseudomonas fluorescensi*	Bacteria	marine tunicates	antibacterial	214
Moiramides B (**276**)	*Pseudomonas fluorescensi*	Bacteria	marine tunicates	antibacterial	214
Moiramides C (**277**)	*Pseudomonas fluorescensi*	Bacteria	marine tunicates	antibacterial	214

Abbreviation: cytotox. = cytotoxitity, vs. = against, mod. = moderate, HTCLs=human tumor cell lines, abs. = absolute, config. = configuration, MRSA = Methicillin-resistant *Staphylococcus aureus,* VRE = Vancomycin-Resistant *Enterococcus*, MRSE= Methicillin Resistant *Staphylococus Epidermidis*, TB = *M.tuberculosis*, NF= not found or no test bioactivities, ND= not detected
